# 17β-Estradiol Treatment Improves Acetylcholine-Induced Relaxation of Mesenteric Arteries in Ovariectomized UC Davis Type 2 Diabetes Mellitus Rats in Prediabetic State

**DOI:** 10.3389/fphys.2022.900813

**Published:** 2022-06-17

**Authors:** Md Rahatullah Razan, Farjana Akther, Rifat A. Islam, James L. Graham, Kimber L. Stanhope, Peter J. Havel, Roshanak Rahimian

**Affiliations:** ^1^ Department of Physiology and Pharmacology, Thomas J. Long School of Pharmacy, University of the Pacific, Stockton, CA, United States; ^2^ Department of Molecular Biosciences, School of Veterinary Medicine, Department of Nutrition, University of California, Davis, Davis, CA, United States

**Keywords:** 17β-estradiol, prediabetes, UC Davis type 2 diabetes mellitus, mesenteric artery, vasorelaxation, nitric oxide

## Abstract

We recently reported sex differences in mesenteric arterial function of the UC Davis type-2 diabetes mellitus (UCD-T2DM) rats as early as the prediabetic state. We reported that mesenteric arteries (MA) from prediabetic male rats exhibited a greater impairment compared to that in prediabetic females. However, when females became diabetic, they exhibited a greater vascular dysfunction than males. Thus, the aim of this study was to investigate whether the female sex hormone, estrogen preserves mesenteric arterial vasorelaxation in UCD-T2DM female rats at an early prediabetic state. Age-matched female Sprague Dawley and prediabetic (PD) UCD-T2DM rats were ovariectomized (OVX) and subcutaneously implanted with either placebo or 17β-estradiol (E_2,_ 1.5 mg) pellets for 45 days. We assessed the contribution of endothelium-derived relaxing factors (EDRF) to acetylcholine (ACh)-induced vasorelaxation, using pharmacological inhibitors. Responses to sodium nitroprusside (SNP) and phenylephrine (PE) were also measured. Additionally, metabolic parameters and expression of some targets associated with vascular and insulin signaling were determined. We demonstrated that the responses to ACh and SNP were severely impaired in the prediabetic state (PD OVX) rats, while E_2_ treatment restored vasorelaxation in the PD OVX + E_2_. Moreover, the responses to PE was significantly enhanced in MA of PD OVX groups, regardless of placebo or E_2_ treatment. Overall, our data suggest that 1) the impairment of ACh responses in PD OVX rats may, in part, result from the elevated contractile responses to PE, loss of contribution of endothelium-dependent hyperpolarization (EDH) to vasorelaxation, and a decreased sensitivity of MA to nitric oxide (NO), and 2) the basis for the protective effects of E_2_ may be partly attributed to the elevation of the NO contribution to vasorelaxation and its interaction with MA as well as potential improvement of insulin signaling. Here, we provide the first evidence of the role of E_2_ in protecting MA from early vascular dysfunction in prediabetic female rats.

## 1 Introduction

The prevalence of type-2 diabetes (T2D) is increasing at an alarming rate worldwide (Zheng et al., 2018). Cardiovascular diseases (CVD) are the primary cause of death and disability in diabetic patients (Einarson et al., 2018). It is now well established that sex differences exist in CVD onset, severity, and outcomes (Miller, 2010). The prevalence of CVD in premenopausal women is less than age-matched men, and female sex hormones, estrogen in particular, play a role in sex-specific cardiovascular protection in females (Barrett-Connor, 1994; Iorga et al., 2017). Several studies including ours suggest that diabetes affects male and female vascular beds differently (Witcher et al., 2010; Zhang et al., 2012; Lum-Naihe et al., 2017; Shaligram et al., 2020; Akther et al., 2021). Diabetes not only abrogates the female-specific cardiovascular protection, but also premenopausal women with diabetes experience higher CVD events than diabetic men (Peters et al., 2015; Castro, 2016), suggesting that hyperglycemia may overcome some of the beneficial effects of female sex hormones. However, the specific role of estrogen and the timeline of the loss of female-specific cardiovascular protection in diabetic premenopausal women are not well understood.

An appropriate animal model may provide critical insights into the pathogenesis of cardiovascular dysfunction in T2D. The current study was performed in mesenteric arteries (MA) of a polygenic rodent model of T2D: UC Davis type-2 diabetes mellitus (UCD-T2DM) rats in the prediabetic state. This model of T2D exhibits polygenic adult-onset obesity, insulin resistance, pancreatic beta-cell decompensation and preserved leptin signaling and fertility (Cummings et al., 2008).

Endothelial dysfunction is considered a critical early sign of CVD. Endothelium-dependent vasorelaxation (EDV) is used as a reproducible parameter to examine endothelial function. Reduced EDV was reported in both type-1 and type-2 diabetes (Zhang et al., 2012; Han et al., 2016; Shaligram et al., 2020). Altered EDV could result from reduced release and/or synthesis of endothelium-derived relaxing factors (EDRF) [nitric oxide (NO), prostacyclin (PGI_2_), and NO- and prostanoid-independent mediators)] and/or increased production of endothelium-derived contractile factors (EDCF).

Contribution of EDRF to EDV may vary based on the vascular bed. While in large conduit arteries, NO is the major contributor for vasorelaxation, in small resistance arteries NO- and prostanoid- independent or endothelium-dependent hyperpolarization (EDH) mediators were found to be the major contributor for EDV (Félétou, 2011; Zhang et al., 2012). Previous reports from our group suggest diabetes alters NO and EDH-type contribution in both large and small resistance arteries (Shaligram et al., 2020; Akther et al., 2021). Estrogen was reported to increase EDH-type relaxation in mesenteric and uterine resistance arteries (Burger et al., 2009). However, the specific role of estrogen on NO and EDH contribution in mesenteric arterial relaxation at the prediabetic state was not investigated.

We recently reported sex differences in mesenteric arterial function of UCD-T2DM rats as early as the prediabetic state (Shaligram et al., 2020). In particular, vascular relaxation to acetylcholine (ACh) was impaired to a greater extent in MA from males in the prediabetic state than in their female counterparts. In contrast, the arteries from females with diabetes exhibited a greater impairment to ACh-induced vasorelaxation compared with diabetic males (Shaligram et al., 2020). Here, we hypothesized that estrogen might be responsible for delaying vascular dysfunction in the prediabetic females. Thus, in the current study, we investigated the effect of 17-β estradiol (E_2_) administration on mesenteric arterial function of ovariectomized rats in the prediabetic state. We provide the first evidence of the specific role of estrogen in protecting MA from early vascular dysfunction in female prediabetic UCD-TDM rats, possibly *via* elevation of NO contribution to vasorelaxation and its interaction with arteries.

## 2 Materials and Methods

### 2.1 Materials

All chemicals were purchased from either Fisher Scientific (Waltham, MA) or Sigma Aldrich (St. Louis, MO, United States) and dissolved in water unless otherwise stated.

### 2.2 Experimental Animals

The UCD-T2DM rats were generated by selectively breeding obese Sprague Dawley (SD) rats with Zucker Diabetic Fatty (ZDF) lean rats at the animal facility in the Department of Nutrition at the University of California, Davis (Cummings et al., 2008).

For the purposes of this study, we selected 13–14-week-old female UCD rats that had not yet developed diabetes (prediabetic group) but had higher body weight than SD control rats. Furthermore, they were considered prediabetic when the non-fasting blood glucose readings of animals were in the range of 150–200 mg/dl (Guglielmino et al., 2012). These prediabetic female rats were then surgically ovariectomized (OVX) and implanted subcutaneously with either 17β-estradiol (E_2_, 1.5 mg/pellet, 60-days uniform release) or placebo pellets (Innovative Research of America, Sarasota, FL). Similarly, age-matched and OVX SD females (control), implanted subcutaneously with E_2_ (1.5 mg/pellet, 60-days uniform release) or placebo pellets were purchased from Charles River Laboratories (Wilmington, MA). Animals were divided into four experimental groups: 1) OVX + placebo [OVX], 2) OVX + E_2_, 3) prediabetic OVX + placebo [PD OVX], 4) prediabetic OVX + E_2_ [PD OVX + E_2_]. Both prediabetic UCD and control rats stayed at their respective facility (UC Davis and Charles Rivers) for 1 week after pellet implantation. The rats were later transferred to animal facility at the University of the Pacific.

All the animals were maintained with ad libitum water and standard rodent chow food (Mazuri rodent chow) at constant humidity and temperature-controlled room with a 12 h light/dark cycle at the University of the Pacific vivarium. Notably, our collaborators at UC Davis performed an OVX study and did not observe much difference in food intake between OVX and OVX + E_2_ groups. The animals were euthanized using carbon dioxide at 45 days after the implantation of the pellets. The euthanization process was completed following AVMA Guidelines for the Euthanasia of Animals: 2013 edition and the NIH Guidelines for the Care and Use of Laboratory Animals: Eighth Edition. All animal protocols were approved by the Institutional Animal Care and Use Committee of the University of the Pacific and UC Davis and complied with the Guidelines for the Care and Use of Laboratory Animals: Eighth Edition (US National Institutes of Health, 2011) and ARRIVE guidelines (Kilkenny et al., 2010). The intra-abdominal adipose tissue was measured by collecting all visceral white adipose tissue located around the digestive organs (mesenteric and omental), after removal of mesenteric arterial cascade. The intra-abdominal adipose tissue weight/total body weight ratio was then calculated, and results were expressed as percentage of intra-abdominal adipose tissue compared to total body weight.

### 2.3 Blood and Plasma Analysis

A drop of blood from the tail vein was used to measure random blood glucose and triglyceride level by hand-held point of care devices. Glucose levels were measured by a standard glucometer (OneTouch UltraMini) and triglyceride levels were measured by Accutrend Plus System (Roche Farma, Barcelona, Spain) using specific strip for each device.

Blood samples were also obtained by intracardiac puncture after euthanizing the animals and collected in tubes containing heparin and sodium citrate. The tubes were then centrifuged at 10,000×g for 5 min at 4°C and plasma was collected and aliquoted into fresh tubes to be stored at −80°C for later analysis. Plasma insulin and E_2_ level were measured using ELISA kits according to the manufacturer’s protocol (Mercodia, Uppsala, Sweden; Abcam, Cambridge, MA). Blood collected by intracardiac puncture was also used for glycated hemoglobin (HbA1c) level analysis using A1cNow kit (PTS diagnostics, Sunnyvale, CA) following the manufacturer’s instructions.

### 2.4 Measurement of Mesenteric Arterial Tension

Third-order mesenteric arterial branches were isolated and cleared off from veins, fat, and other surrounding tissues and were precisely cut into 2 mm rings. Each ring was then mounted in an organ bath, between the two jaws of a wire myograph (model 610M, Danish Myo Technology (DMT), Denmark) with the help of two tungsten wires (diameter 40 μm). The organ bath contained Krebs solution of (in mM) 119 NaCl, 4.7 KCl, 1.6 CaCl_2_, 1.2 MgSO_4_, 1.2 KH_2_PO_4_, 25 NaHCO_3_, 0.023 EDTA, and 6 glucose at 37°C, bubbled with 95% O_2_–5% CO_2_. A computer-based data acquisition system was used to monitor the variation of arterial isometric tension (Labchart version 7.3.8, Powerlab; ADInstruments, Colorado Springs, CO).

The arteries were normalized to a resting passive pressure of 13.3 kPa by using a built-in normalization module in wire myograph, according to the guideline provided by DMT (DMT manual) and published reports (Bridges et al., 2011; Wenceslau et al., 2021). The micrometer was gradually increased until approximately 13.3 kPa/100 mmHg pressure was achieved. It is important to note that the micrometer was not moved back after reaching 13.3 kPa, thus our normalization factor was close to 1.0.

The tissues were then equilibrated for 30 min to obtain a basal tone, before 80 mM KCl solution was used twice for a short period to stimulate the arterial segments. Subsequently, ACh (10 µM) induced relaxation was recorded in phenylephrine (PE, 2 µM) precontracted arteries to evaluate the viability of the endothelium. Drugs were rinsed out, the vessels were re-equilibrated for 30 min, and a cumulative concentration-response curve (CRC) to PE (10^−8^ to 10^−5^ M) was performed. For the vasorelaxation studies, the arteries were precontracted with 2 µM of PE which induced approximately 80% of contraction achieved by 10 μM of PE (Supplementary Table S1).

#### 2.4.1 Relaxation Responses to ACh

Increasing concentrations of ACh (10^−8^ to 10^−5^ M) were added to PE (2 µM) precontracted artery rings to obtain the concentration-response curve (CRC).

The vascular relaxation to ACh (10^−8^ to 10^−5^ M) in mesenteric arterial rings were then obtained after pretreatment with indomethacin [Indo, 10µM, a blocker of the cyclooxygenase (COX)], followed by addition of L-NAME [200µM, nitric oxide synthase (NOS) blocker] and then a combination of apamin [1µM, small conductance calcium activated potassium channel (SK_Ca_) inhibitor] and TRAM-34 [1µM, intermediate conductance calcium activated potassium channel (IK_Ca_) inhibitor]. A representative trace of time control for the CRC to ACh (10^−8^–10^−5^ M) as well as the sensitivity and maximum relaxation response to ACh have been shown in Supplementary Figure S1. However, the sample size for the time control study was small (n = 4).

#### 2.4.2 Relaxation Responses to Sodium Nitroprusside (SNP)

The CRC to SNP (10^−9^ to 10^−5^ M) was obtained in intact MA precontracted with PE (2 µM) in the absence of any pharmacological inhibitors.

#### 2.4.3 Contractile Responses to PE and Endothelin-1 (ET-1) 

The CRC to PE and ET-1 (Tocris Biosciences, Minneapolis, MN) were obtained by the addition of increasing concentrations of PE (10^–8^ to 10^–5^ M) or ET-1 (10^−10^ to 10^−7^ M) to the myograph chamber. The concentration of drugs used to generate relaxation or contraction curves were based on our previous reports (Zhang et al., 2012; Han et al., 2014, 2016; Shaligram et al., 2020).

### 2.5 Western Blot Analysis

All tissue samples harvested after euthanizing the animals were flash frozen by liquid nitrogen and saved in −80°C for later analysis. MA and skeletal muscle (SKM) samples were micronized using gentleMACs tissue dissociator (Miltenyi Biotech, Bergisch, Germany), following manufacturer’s protocol for protein extraction. Commercial RIPA buffer supplemented with phosphatase and protease inhibitor cocktail (ThermoFisher Scientific, Waltham, MA) were used to obtain the total protein extract from the tissues. Briefly, for processing by gentleMACs tissue dissociator, tissues were placed in M-tubes (Miltenyi Biotech, Bergisch, Germany) containing RIPA buffer, phosphatase, and protease inhibitor cocktail. Protein extraction protocol was selected from menu and after 1 min the blended tissue extract was centrifuged at 15,000×g for 15 min at 4°C, and supernatants were collected. Total protein concentration of the extract was determined by BCA gold assay (ThermoFisher Scientific, Waltham, MA).

20–30 µg of protein for each sample was loaded in the sodium dodecyl sulfate poly acrylamide gels (SDS-PAGE) and subjected to gel electrophoresis. Protein was then transferred to a 0.45 μm nitrocellulose membranes (Bio Rad Laboratories Inc, Hercules, CA), blocked for 1 h at room temperature with 5% w/v BSA in 0.1% Tween 20-Tris-buffered saline (TBS), and incubated overnight at 4°C with primary antibodies similarly as described by us (Akther et al., 2021). Primary antibodies for endothelial nitric oxide synthase (eNOS) (#32027), AMP-activated protein kinase-α (AMPK-α) (#2532) and phospho-AMP-activated protein kinase-α (pAMPK-α, Thr-172) (#2535), insulin receptor substrate 1 (IRS1) (#2390) were obtained from Cell Signaling Technology (Danvers, MA). Antibodies against NADPH oxidase-1 (NOX-1) (#ab131088) and glucose transporter-4 (GLUT-4) (#ab654) were obtained from Abcam (Cambridge, MA). All primary antibodies were diluted to 1:1,000 except for IRS1, which was diluted to 1:500. After incubation with primary antibodies the membranes were washed 4 times and incubated 1 h at room temperature with IRDye 680 Donkey anti-Rabbit IgG secondary antibody (dilution 1:10,000, LI-COR, Lincoln, NE). Finally, after removing secondary antibody, membranes were washed 4 times with TBS containing 0.1% Tween-20 and bands were detected using LICOR Odyssey imaging system. The bands were quantified by densitometry using LI-COR Image Studio Lite software. To confirm the uniformity of protein loading, blots were incubated with GAPDH antibodies (#2118, Cell Signaling Technology), normalized to GAPDH level and expressed as fold changes from control E_2_ treated group.

### 2.6 Statistical Analysis

Vasorelaxation to ACh and SNP were expressed as percent relaxation response from maximum PE (2 µM) contraction at each concentration. The concentration that produces half of the maximum relaxation (EC_50_) was calculated by sigmoidal concentration response model with variable slope by GraphPad Prism 8.0 (GraphPad Software, San Diego, CA) and expressed as sensitivity to the agonist; pD_2_ values (-logEC_50_). Maximum relaxation response to agonist was expressed as R_max_ and maximum tension to contractile agent such as PE was expressed as Tension_max_. One way ANOVA was used to compare the means between different groups (i.e., EC_50_, R_max_, blood glucose level). When one-way ANOVA returned *p* < 0.05, Tukey’s post-hoc test was used to obtain the groups which were different from each other. Comparison of CRCs between different groups were analyzed using two-way ANOVA with repeated measure, with concentration considered as repeated measure, followed by Tukey’s post-hoc test. To compare the CRC before and after treatment with drugs within a group, two-way ANOVA with repeated measure followed by Bonferroni’s post hoc analysis was used. Statistical analysis of protein expression was performed by one-way ANOVA and Tukey’s post-hoc test.

## 3 Results

### 3.1 Effects of E_2_ Treatment on Metabolic Parameters

**FIGURE 1:**
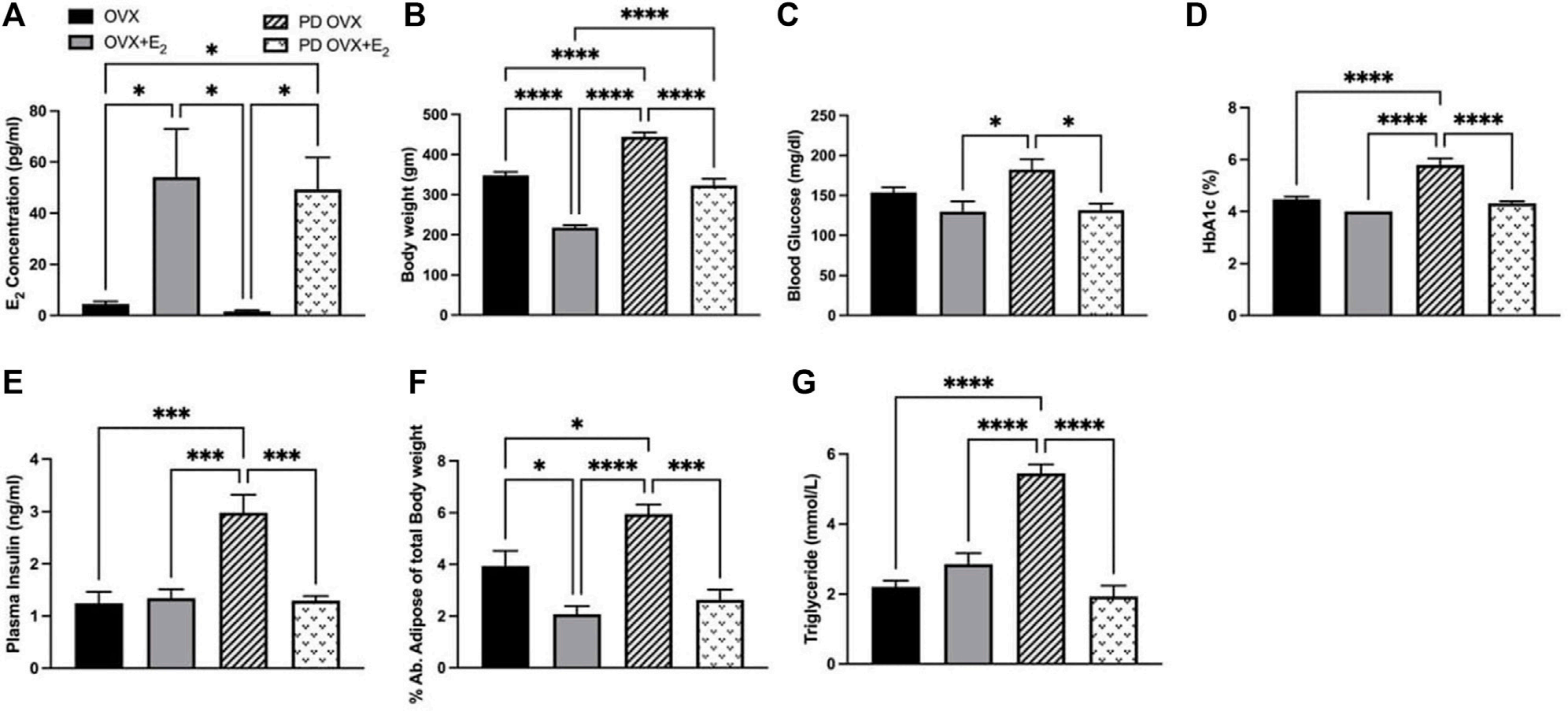
Metabolic parameters of ovariectomized (OVX) and E_2_ replaced (OVX+E_2_) control and prediabetic (PD) rats. **(A)** Plasma estradiol (E_2_) concentration **(B)** Body weight **(C)** Blood glucose level **(D)** HbA1c **(E)** Plasma insulin level **(F)** Abdominal adipose tissue percentage to total body weight **(G)** Circulating triglyceride level. Values are represented as mean ± SEM. Each bar represents the values obtained from *n* = 5–6 animals per group. Capped lines indicate significant differences between two groups (^*^ *p* < 0.05, ^***^ *p* < 0.001, ^****^ *p* = 0.0001), analyzed using one-way ANOVA followed by Tukey’s post hoc test.

E_2_ treatment significantly increased the concentrations of plasma E_2_ in OVX groups, both in OVX + E_2_ and PD OVX + E_2_ groups (Figure 1A). In particular, plasma E_2_ concentrations were significantly lower in OVX, and PD OVX rats compared with those in respective E_2_-treated groups. In agreement with Ferrer et al., 1996 and our previous report (Ferrer et al., 1996; Rahimian et al., 1997b), the body weights of E_2_-treated rats were significantly lower than those of OVX rats, regardless of being OVX + E_2_ or PD OVX + E_2_ (Figure 1B). Similar to body weight, percent of intra-abdominal adipose tissue (located around mesentery and omental) was significantly lower in OVX + E_2_ and PD OVX + E_2_ groups compared to their respective controls (Figure 1F). When compared to E_2_-treated groups, PD OVX group had significantly higher glucose level (Figure 1C). Accordingly, HbA1c, plasma insulin, and triglyceride levels were significantly higher in PD OVX group compared to all other experimental groups (Figures 1D,E,G).

### 3.2 Effects of E_2_ Treatment on IRS1 and GLUT-4 Expressions in SKM

**FIGURE 2:**
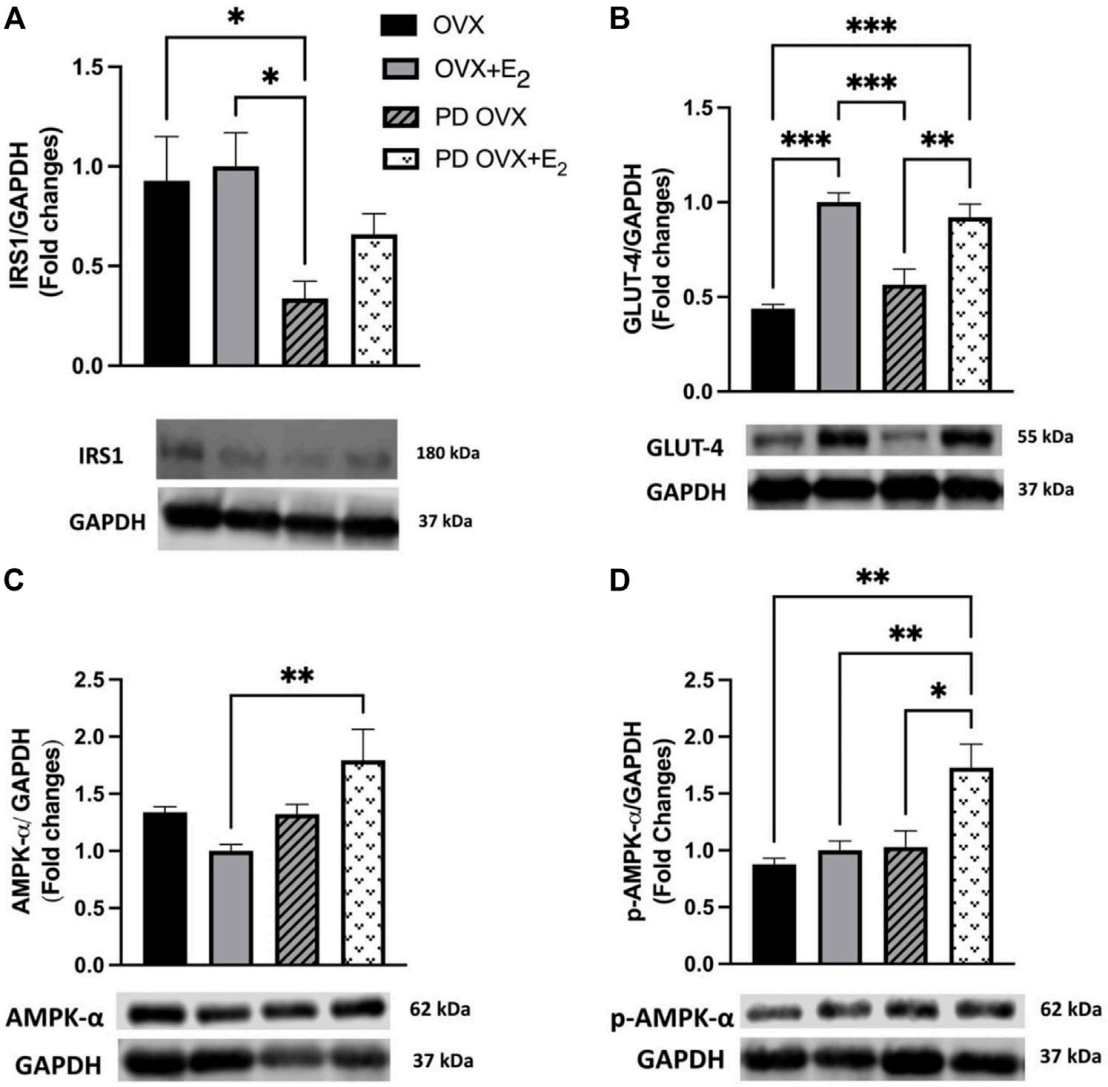
Western blot analysis of **(A)** IRS1 **(B)** GLUT-4 **(C)** AMPK-α **(D)** p-AMPK-α expression in skeletal muscle of ovariectomized (OVX) and E_2_ replaced (OVX+E_2_) control and prediabetic (PD) rats. Protein level was quantified by densitometry and normalized to corresponding GAPDH. Values are represented as mean ± SEM. Each bar represents the values obtained from *n* = 4–5 animals per group. Representative bands of target and housekeeping protein (GAPDH) were shown from the same membranes. Capped lines indicate significant differences between two groups (^*^ *p* < 0.05, ^**^ *p* < 0.01, ^***^ *p* < 0.001), analyzed using one-way ANOVA followed by Tukey’s post hoc test. IRS1, insulin receptor substrate 1; GLUT-4, glucose transporter-4; AMPK-α, AMP-activated protein kinase-α; p-AMPK-α, phosphorylated-AMP-activated protein kinase-α.

The SKM is the major site for insulin-mediated glucose uptake. Although β-cell failure is a common characteristic of T2D, SKM insulin resistance and reduced glucose clearance are considered the initiating factors that lead to overt hyperglycemia and β-cell dysfunction (Warram et al., 1990; DeFronzo and Tripathy, 2009). Higher glucose and HbA1c levels together with higher insulin level (Figures 1C–E) in the PD OVX group prompted us to analyze the expression of the main insulin signal transducer, IRS1 and GLUT-4 in SKM. As shown in Figure 2A, IRS1 expression in SKM was significantly lower in PD OVX group compared to OVX and OVX + E_2_ groups. There was no difference in IRS1 expression in SKM of OVX and OVX + E_2_ or PD OVX and PD OVX + E_2_ (However, this could be due to small sample size per group used for this comparison). When GLUT-4 expression was investigated, both PD OVX and OVX groups showed significantly lower expression compared to respective E_2_-treated group.

Previous reports also indicate that E_2_ may activate AMPK-α leading to an increase in glucose uptake in SKM in C57 BL/6 mice (D’Eon et al., 2005). Thus, we investigated whether AMPK-α and p-AMPK-α levels in SKM were altered in E_2_-treated groups. E_2_ treatment significantly increased AMPK-α and p-AMPK-α levels in SKM of PD OVX + E_2_ (Figures 2C,D). When compared the GAPDH level among experimental groups, there was no significant change in GAPDH level between groups (Data not shown).

### 3.3 Effects of E_2_ Treatment on Relaxation Responses to ACh

**FIGURE 3:**
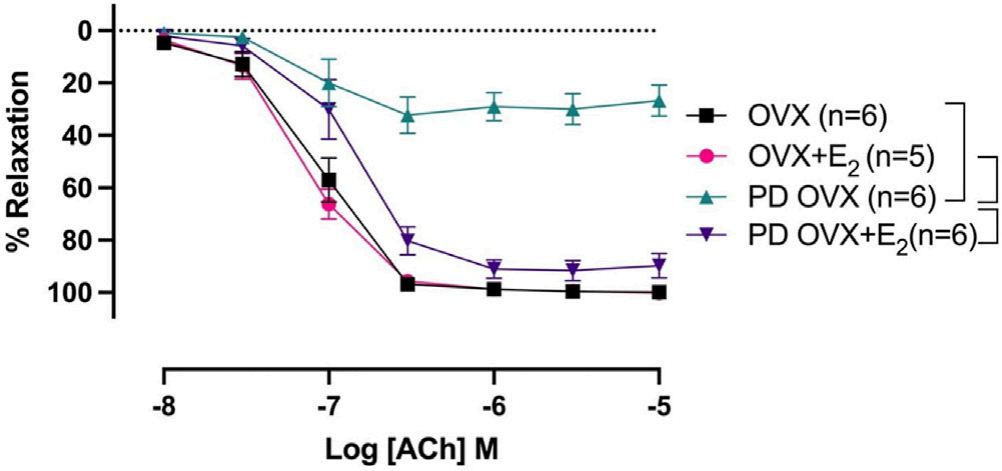
Concentration-response curve (CRC) to acetylcholine (ACh, 10^−8^ to 10^−5^ M) in intact mesenteric arterial rings precontracted with phenylephrine (PE, 2 μM) from ovariectomized (OVX) and E_2_ replaced (OVX+E_2_) control and prediabetic (PD) rats. Data are expressed as mean ± SEM. *n* = 5–6 animals per group. Brackets indicate significant differences between groups (*p* < 0.05), analyzed using 2-way ANOVA with repeated measures followed by Tukey’s post hoc test.

ACh (10^−8^ –10^−5^ M) concentration dependently relaxed PE-precontracted mesenteric arterial rings. There were no significant differences in ACh-induced relaxation in MA from OVX rats compared with those from OVX + E_2_ rats, as indicated by no significant differences between R_max_ and pD_2_ values of the OVX and OVX + E_2_ CRC to ACh (Figure 3; Table 1). The ACh CRC was markedly impaired in MA of PD OVX group compared to those in OVX and OVX + E_2_ groups (Figure 3). Both R_max_ and pD_2_ values were significantly lower in the MA of PD OVX group when compared to OVX and OVX + E_2_ groups (Table 1). However, E_2_ treatment significantly enhanced the R_max_ and pD_2_ to ACh in the PD OVX + E_2_ rats compared to the PD OVX group (Figure 3; Table 1). In a supplemental study, we included an additional sham-operated PD female group to determine whether the impairment of ACh in PD OVX occurred as a result of ovariectomy. As shown in Supplementary Figure S2, the ACh-induced vasorelaxation in MA of sham-operated PD group were significantly higher than that in PD OVX rats, as assessed by pD_2_ and R_max_.

**TABLE 1:** Sensitivity (pD_2_: logEC_50_), and Maximum Response (R_max_) to acetylcholine (ACh) in mesenteric arteries from ovariectomized (OVX) and E_2_ treated (OVX + E_2_) control and prediabetic (PD) rats.

ACh	n	pD_2_ (-logEC_50_)	R_max_ (%)
OVX	6	7.10 ± 0.05	99.75 ± 0.27
OVX + E_2_	5	7.14 ± 0.03	100.1 ± 0.1
PD OVX	6	2.53 ± 0.9^#*^	41.77 ± 5.66^#*^
PD OVX + E_2_	6	6.82 ± 0.07^$^	91.06 ± 3.93^$^

Data are expressed as mean ± SEM; n = 5-6 animals per group. ^#^ *p* < 0.05 (vs. OVX), ^*^ *p* < 0.05 (vs. OVX + E2), ^$^ *p* < 0.05 (vs. PD OVX), analyzed using one-way ANOVA, followed by Tukey’s post-hoc test.

### 3.4 Effects of E_2_ Treatment on the Relative Contribution of NO to Relaxation Responses to ACh

**FIGURE 4:**
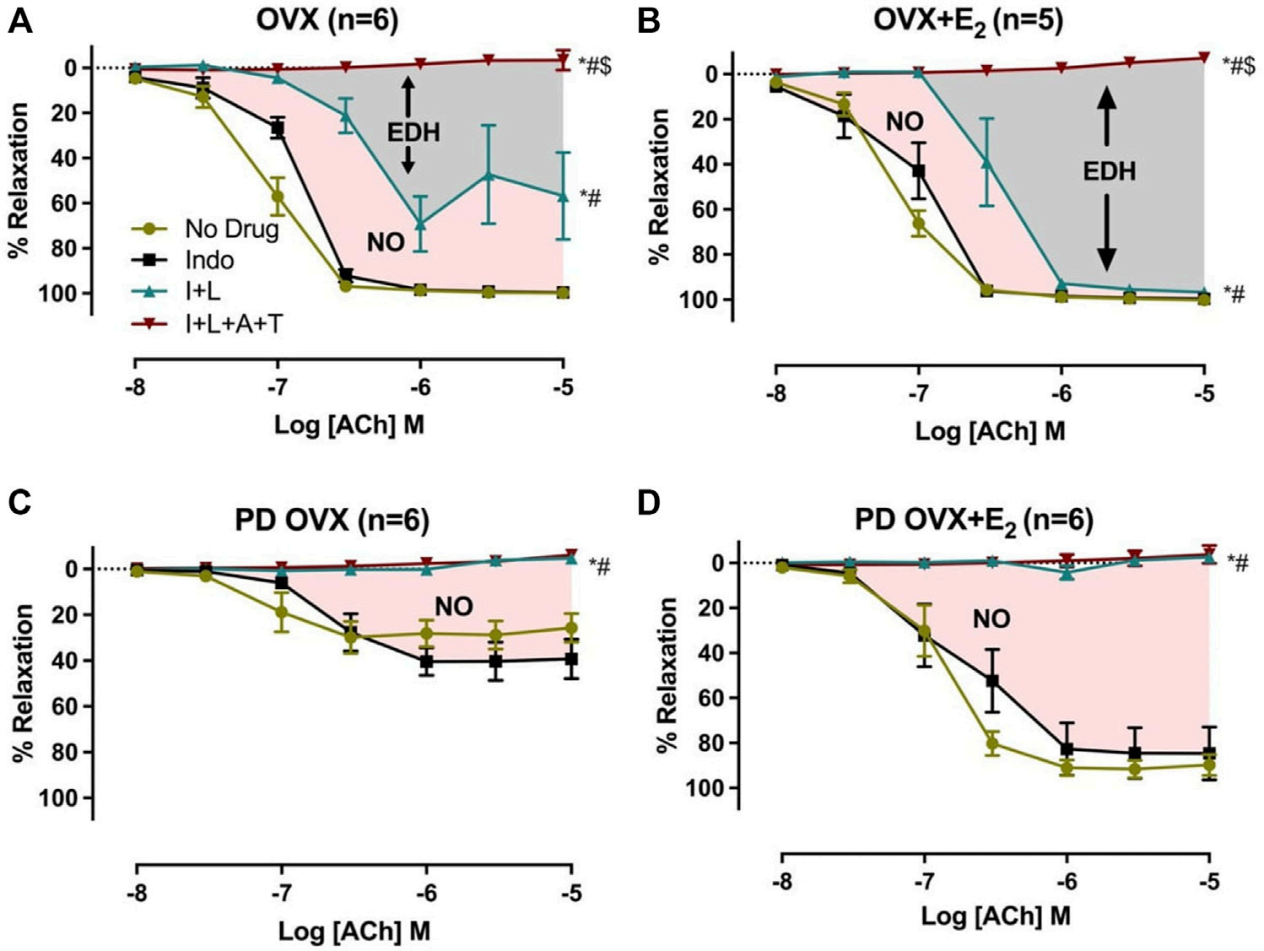
Effects of inhibiting cyclooxygenase (COX), nitric oxide synthase (NOS), small conductance and intermediated conductance calcium activated potassium channels (SKca and IKca) on acetylcholine (ACh)-induced vasorelaxation in mesenteric arteries taken from ovariectomized (OVX) and E_2_ replaced (OVX+E_2_) control and prediabetic (PD) rats; **(A)** OVX **(B)** OVX+E_2_ **(C)** PD OVX **(D)** PD OVX+E_2_. Concentration response curve (CRC) to ACh (10^−8^ to 10^−5^) was generated in the absence (No drug) or in the presence of indomethacin (Indo), Indo+L-NAME (I+L), and Indo+L-NAME+Apamin+TRAM-34 (I+L+A+T) in mesenteric arterial rings. Indo (indomethacin; 10μM), L-NAME (N^ω^-nitro-L-arginine methyl ester; 200 μM), Apamin (1 μM), TRAM-34; [1-{(2-Chlorophenyl) diphenylmethyl}-1H-pyrazole; 1 μM]. Data are expressed as mean ± SEM. *n* = 5–6 animals per group. * (P < 0.05) vs. no drug; # (P < 0.05) vs. Indo; $ (P < 0.05) vs. Indo+L-NAME, analyzed using 2-way ANOVA repeated measures followed by Bonferroni’s post hoc test. NO, nitric oxide; EDH, endothelium-dependent hyperpolarization.

The relative contribution of PGI_2_, NO, and EDH-mediators to vasorelaxation induced by ACh was determined by sequentially blocking COX, NOS, and a combination of SK_Ca_ and IK_Ca_ channels, as previously reported by others and us (Sunano et al., 1999; Matchkov et al., 2012; Zhang et al., 2012; Shaligram et al., 2020). Specifically, EDV to ACh (10^−8^ to 10^−5^ M) in rat mesenteric arterial rings precontracted with PE (2 µM) was obtained before and after pretreatment with Indo (10 µM), followed by addition of L-NAME (200 µM) and then a combination of apamin (1 µM) and TRAM-34 (1 µM). The presence of Indo did not significantly change the ACh CRC in any of the groups when compared to no drug ACh CRC (Figures 4A–D). Addition of L-NAME resulted in a significant reduction of the ACh relaxation in OVX and OVX + E_2_ groups but did not completely block the relaxation. After the addition of L-NAME, the R_max_ to ACh was 75.3 ± 9.4% in OVX and 96.7 ± 1.4% in OVX + E_2_ rats (Table 2). The remaining vasorelaxation to ACh after addition of L-NAME in the OVX and OVX + E_2_ groups suggesting a role of prostanoid- and NO-independent relaxation responses in these groups. Finally, pretreatment of MA with apamin and TRAM-34 in the presence of Indo and L-NAME completely abolished the remaining ACh-induced vasorelaxation in these groups (Figures 4A,B).

**TABLE 2:** Sensitivity (pD_2_: logEC_50_), Maximum Response (R_max_), and Area Under the Curve (AUC) to acetylcholine (ACh) in mesenteric arteries from ovariectomized (OVX) and E_2_ replaced (OVX + E_2_) control and prediabetic (PD) rats.

Groups	No drug			Indo			Indo + L-NAME			Indo + L-NAME + Apamin + TRAM-34		
	pD_2_	R_max,_ (%)	ΔAUC	pD_2_	R_max_ (%)	ΔAUC	pD_2_	R_max_ (%)	ΔAUC	pD_2_	R_max_ (%)	ΔAUC
OVX	7.10 ± 0.05	99.75 ± 0.27	ND	6.88 ± 0.1^a^	99.65 ± 0.3	20.1 ± 10.2	ND	75.3 ± 9.4^ab^	103.5 ± 26.3	ND	1.73 ± 3.7^abc^	86.0 ± 25.6
OVX + E_2_	7.14 ± 0.03	100.1 ± 0.1	ND	7.06 ± 0.1	99.56 ± 0.2^a^	8.8 ± 13.7	ND	96.7 ± 1.4^#^	65.4 ± 19.7	ND	-1.91 ± 2.9^abc^	139.6 ± 15.3
PD OVX	2.53 ± 0.9^#*^	41.77 ± 5.66^#*^	ND	4.56 ± 0.5^#*^	50.68 ± 6.6^#*^	6.86 ± 17.6	ND	1.88 ± 1.1^#*ab^	64.4 ± 12.6	ND	-2.41 ± 1.0^abc^	3.7 ± 1.7^#*^
PD OVX + E_2_	6.82 ± 0.07^$^	91.06 ± 3.93^$^	ND	6.30 ± 1.1^$^	85.0 ± 11.3^$^	22.8 ± 26.2	ND	4.23 ± 2.9^#*ab^	147.0 ± 12.2^*$^	ND	-1.0 ± 2.7^ab^	3.5 ± 3.2^#*^

A comparison of sensitivity (pD_2_: logEC_50_), maximum response (R_max_) and ΔAUC, to acetylcholine (ACh) in the absence (No drug) or in the presence of Indomethacin (Indo), Indo + L-NAME, and Indo + L-NAME + Apamin + TRAM-34, in mesenteric arterial rings taken from ovariectomized (OVX) and E_2_ replaced (OVX + E_2_) control and prediabetic (PD) rats. ΔAUC, was measured by determining the area between two curves; ΔAUC, of Indo indicates cyclooxygenase (COX) contribution, ΔAUC, of Indo + L-NAME, indicates nitric oxide (NO) contribution and, ΔAUC, of Indo + L-NAME + Apamin + TRAM-34, indicates endothelium-dependent hyperpolarization (EDH)-type contribution in ACh-induced vasorelaxation of mesenteric artery. Data are expressed as mean ± SEM; n = 5-6 rats per group. Analysis between group: ^#^ *p* < 0.05 (vs. OVX), *p < 0.05 (vs. OVX + E_2_), ^$^ *p* < 0.05 (vs. PD OVX), analyzed using one-way ANOVA, followed by Tukey’s post-hoc test. Analysis within group: ^a^p<0.05 vs. No drug control within each group, ^b^p<0.05 vs. Indo within each group, ^c^p<0.05 vs. Indo + L-NAME, within each group (paired Student’s t-test). ND, not determined.

On the other hand, after L-NAME, the ACh-induced vasorelaxation was completely abolished in the PD groups, regardless of E_2_ treatment (Figures 4C,D). However, the effect of L-NAME was more prominent in PD OVX + E_2_ group compared with its respective PD OVX. Specifically, ΔAUC after addition of L-NAME in the PD OVX + E_2_ group was significantly different from the PD OVX group, suggesting an elevated role of NO-dependent relaxation responses in this group (Table 2, fourth column, Figure 4D, Pink shaded area). The ΔAUC after addition of L-NAME in the PD OVX + E_2_ and sham-operated PD groups were 147.0 ± 12.2 and 157.70 ± 9.66, respectively (Supplementary Figures S3D, E). When unpaired *t*-test was used to compare the means, there was no significant difference (*p* = 0.5478). We further assessed as whether two ΔAUC values were equivalent. Statistical analysis revealed the means were not equivalent when the differences between means were selected 20 or less for equivalency analysis (90% confidence interval -21.01–42.42).

### 3.5 Effects of E_2_ Treatment on SNP-Induced Relaxation

**FIGURE 5:**
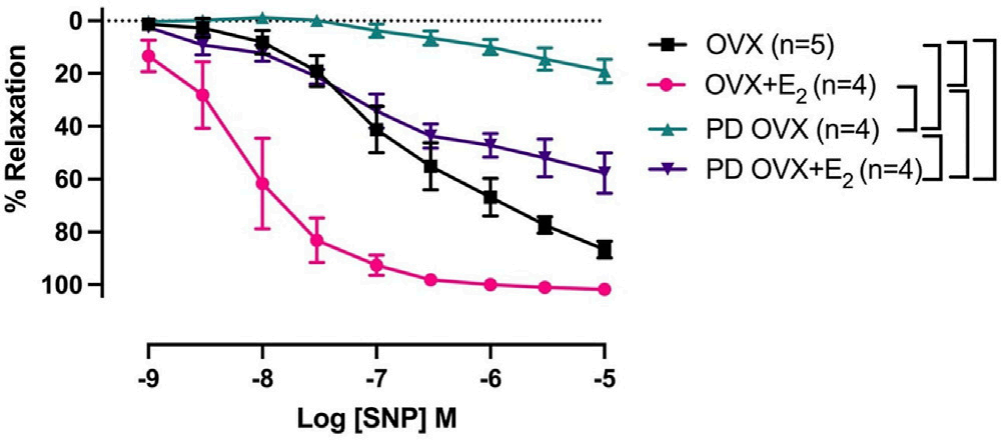
Concentration-response curve (CRC) to sodium nitroprusside (SNP, 10^−9^ to 10^−5^ M) in intact mesenteric arterial rings precontracted with phenylephrine (PE, 2 μM) from ovariectomized (OVX) and E_2_ replaced (OVX+E_2_) control and prediabetic (PD) rats. Data are expressed as mean α SEM. *n* = 4–5 per group. Brackets indicate significant differences (*p* < 0.05) between groups, analyzed using 2-way ANOVA with repeated measures followed by Tukey’s post hoc test.

The mesenteric arterial sensitivity to NO was assessed by measuring the relaxation responses to increasing concentration of SNP (10^−9^ to 10^−5^ M) in intact vessel. Similar to the effects on ACh-induced relaxation, SNP-induced relaxation was significantly impaired in PD OVX compared to that in MA of OVX group. The pD_2_ values to SNP was 6.59 ± 0.2 in OVX, and 3.67 ± 0.2 in PD OVX animals. The R_max_ to SNP in OVX and PD OVX animals were 86.6 ± 3.0% and 19.12 ± 4.3%, respectively. E_2_ treatment enhanced the pD_2_ and R_max_ to SNP in PD OVX + E_2_ rats (Figure 5; Table 3). When compared to OVX group, OVX + E_2_ group exhibited increased pD_2_ (but not a change in R_max_) to SNP-induced relaxation. However, there was still a significant, rightward shift of SNP CRC in MA from PD OVX + E_2_ rats relative to OVX + E_2_ (Figure 5), as assessed by both decreased pD_2_ values and R_max_. The pD_2_ values to SNP was 8.17 ± 0.2 in OVX + E_2_, and 5.49 ± 0.6 in PD OVX + E_2_ animals. The R_max_ to SNP in OVX + E_2_ and PD OVX + E_2_ animals were 101.7 ± 0.6% and 57.68 ± 7.5%, respectively. There was no significant difference in the pD_2_ and R_max_ to SNP-induced relaxation between PD OVX + E_2_ and sham-operated PD rats (Supplementary Figure S4).

**TABLE 3:** Sensitivity (pD_2_: logEC_50_), and Maximum Response (R_max_) to sodium nitroprusside (SNP) in intact rat mesenteric arterial rings from ovariectomized (OVX) and E_2_ replaced (OVX + E_2_) control and prediabetic (PD) rats.

SNP	n	pD_2_	R_max_ (%)
OVX	5	6.59 ± 0.2	86.6 ± 3.0
OVX + E_2_	4	8.17 ± 0.2^#^	101.7 ± 0.6
PD OVX	4	3.67 ± 0.2^#*^	19.12 ± 4.3^#*^
PD OVX + E_2_	4	5.49 ± 0.6^*$^	57.68 ± 7.5^#*$^

Data are expressed as mean ± SEM; n = 4-5 rats per group. ^#^ *p* < 0.05 (vs. OVX), ^*^ *p* < 0.05 (vs. OVX + E_2_), ^$^ *p* < 0.05 (vs. PD OVX), analyzed using one-way ANOVA, followed by Tukey’s post-hoc test.

### 3.6 Effects of E_2_ Treatment on PE-Induced Contraction

**FIGURE 6:**
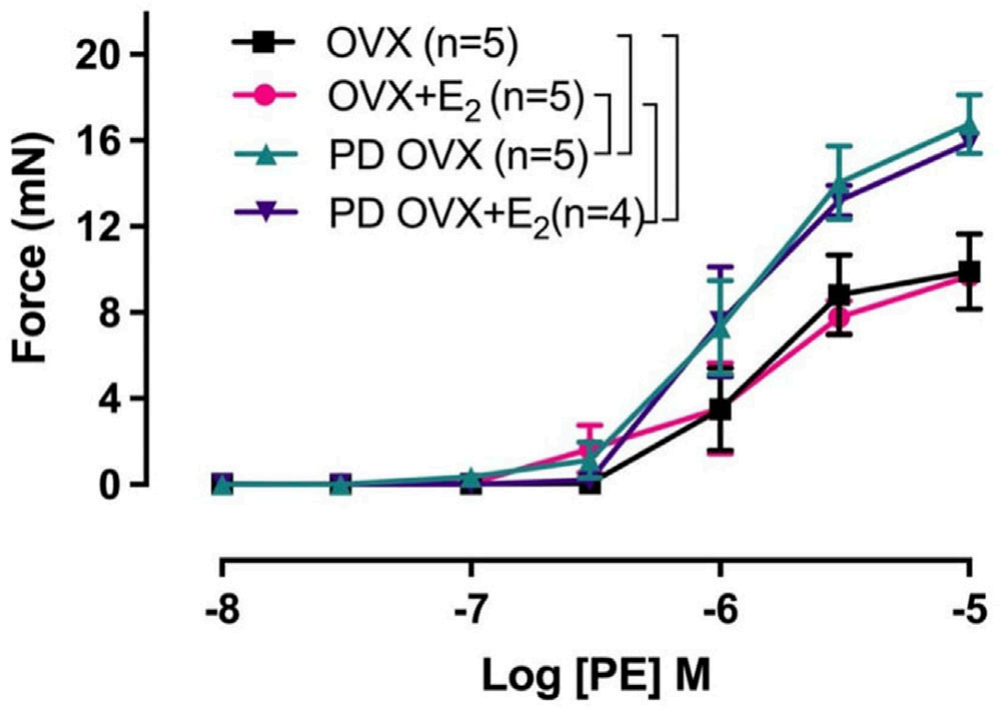
Concentration-response curve (CRC) to phenylephrine (PE, 10^−8^ to 10^−5^ M) in intact mesenteric arterial rings of ovariectomized (OVX) and E_2_ replaced (OVX+E_2_) control and prediabetic (PD) rats. Data are expressed as mean ± SEM. *n* = 4–5 per group. Brackets indicate significant differences between groups (*p* < 0.05), analyzed using two-way ANOVA with repeated measures followed by Tukey’s post hoc test.

Next, we examined whether an elevated contractile response of the arteries was responsible for reduced ACh and SNP-induced relaxation in PD OVX group. We also determined the specific effects of E_2_ treatment on contractile responses in OVX groups. The CRC to PE (10^−8^ to 10^−5^ M) was, therefore, compared in MA among experimental groups (Figure 6). As shown in Figure 6, the maximum contractile response to PE (Tension_max_), but not the sensitivity to PE, was higher in MA of PD OVX and PD OVX + E_2_ when compared to those in OVX and OVX + E_2_ (Figure 6; Table 4). Although, the Tension_max_ were significantly higher in PD vessels, regardless of presence or absence of E_2_, than those in normoglycemic vessels, there were no significant differences in PE sensitivity (pD_2_) and Tension_max_ within control groups (OVX and OVX + E_2_) or within PD groups (PD OVX and PD OVX + E_2_) (Table 4). It is important to note that the differences were seen in PE responses were also observed when another contractile agent, ET-1, was used in the four experimental groups (Supplementary Figure S5).

**TABLE 4:** Sensitivity (pD_2_: logEC_50_), and Tension_max_ to phenylephrine (PE) in rat mesenteric arterial rings from ovariectomized (OVX) and E_2_ replaced (OVX + E_2_) control and prediabetic (PD) rats.

PE	n	pD_2_	Tension_max_ (mN)
OVX	5	6.02 ± 0.1	9.91 ± 1.8
OVX + E_2_	5	5.93 ± 0.2	9.75 ± 0.5
PD OVX	5	5.92 ± 0.1	16.75 ± 1.3^#*^
PD OVX + E_2_	4	5.96 ± 0.1	15.91 ± 0.4^#*^

Data are expressed as mean ± SEM; n = 4-5 rats per group. ^#^ *p* < 0.05 (vs. OVX), ^*^ *p* < 0.05 (vs. OVX + E_2_), analyzed using one-way ANOVA, followed by Tukey’s post-hoc test.

### 3.7 Effects of E_2_ Treatment on eNOS and NOX-1 Expression

**FIGURE 7:**
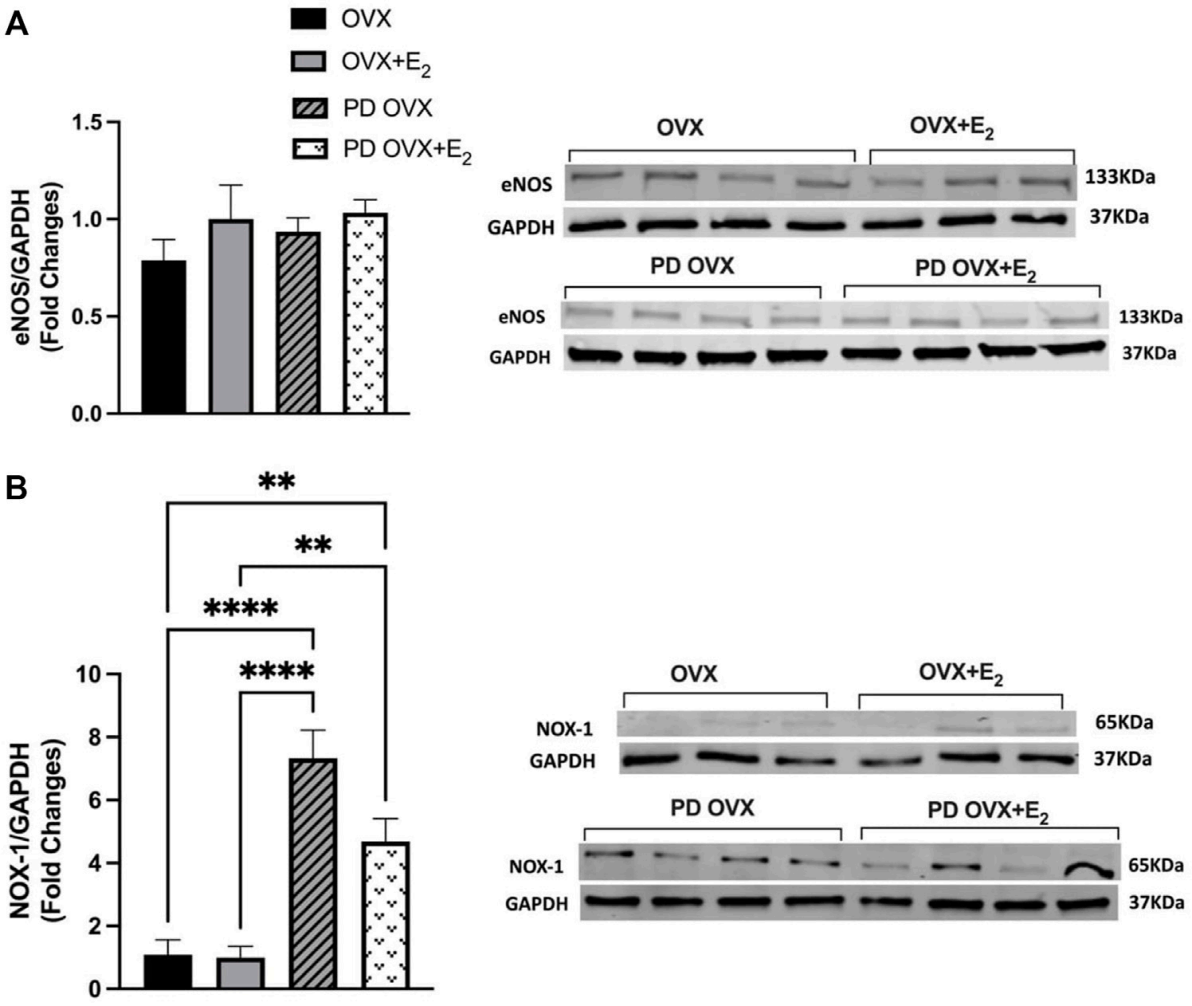
Western blot analysis of **(A)** eNOS and **(B)** NOX-1 expression in mesenteric arteries of ovariectomized (OVX) and E2 replaced (OVX+E_2_) control and prediabetic (PD) rats. Protein levels were quantified by densitometry and normalized to corresponding GAPDH. Values are represented as mean ± SEM. Each bar represents the values obtained from n = 3–4 animals per group. Representative bands of target and housekeeping protein (GAPDH) were shown from the same membranes. Capped lines indicate significant differences between two groups (^**^ *p* < 0.01, ^****^ *p* < 0.0001), analyzed using one-way ANOVA followed by Tukey’s post hoc test. eNOS, endothelial nitric oxide synthase; NOX-1, NADPH oxidase-1.

To investigate the possible mechanisms underlying the elevated contractile responses (or decreased ACh responses) in MA of PD OVX rats, the protein expressions of eNOS as well as NOX-1, a source of superoxide in microvessels and a key driver of oxidative stress within an aberrant metabolic state (Thompson et al., 2017), were measured. Western blot analysis revealed that the expression of eNOS showed no significant difference between the PD OVX group and the OVX group, regardless of E_2_ treatment (Figure 7A). However, as shown in Figure 7B, NOX-1 expression was significantly elevated in MA from PD group, ∼6 fold in PD OVX and ∼3.5 fold in PD OVX + E_2_ compared with those in OVX and OVX + E_2_, respectively.

## 4 Discussion

In a previous work, we reported that vascular relaxation to ACh was impaired to a greater extent in MA from males in the prediabetic state than in their female counterparts. In contrast, the arteries from females with diabetes exhibited reduced vasorelaxation compared with diabetic males (Shaligram et al., 2020). The greater impairment of vasorelaxation in prediabetic males than female arteries suggests that female sex hormones may protect prediabetic females against more severe vascular dysfunction. Here, we provide data that reinforces this hypothesis. To our knowledge, this is the first evidence of the specific role of estrogen in protecting MA from early vascular dysfunction in the prediabetic female UCD-T2DM rats.

It is well known that an increased fat and body mass occur as a result of ovariectomy (Laudenslager et al., 1980; Richard et al., 1987; Latour et al., 2001; Rachoń et al., 2007). Accordingly, both OVX and PD OVX groups in this study exhibited significantly higher body weight and intra-abdominal fat compared to their respective E_2_-treated groups. Previous studies have shown that E_2_ replacement in OVX rodents decreases fat accumulation, improves serum lipid profiles (Shinoda et al., 2002; Wu et al., 2004), and restores insulin action in muscle (Puah and Bailey, 1985; Kumagai et al., 1993). Along similar lines, Chen et al., observed that E_2_ played a role in the regulation of body weight, and a loss of E_2_ was associated with the development of obesity (Chen et al., 2009), which was suggested to be linked to a decrease in serum adiponectin levels (Ritland et al., 2008). In the current study, we did not measure serum adiponectin level, however, when glucose and HbA1c levels were compared among the experimental groups, the PD OVX + E_2_ group exhibited significantly lower glucose and HBA1c levels compared to PD OVX group.

Prior to overt metabolic disorder, abnormally elevated lipid level and hyperinsulinemia are reported (Erion and Corkey, 2017). Here, we observed a significant high level of insulin and triglyceride in PD OVX compared to all other experimental groups. However, E_2_ treatment reduced plasma insulin and triglyceride levels in PD OVX + E_2_.

Insulin resistance is an important characteristic in the pathogenesis of T2D. SKM is the primary organ which under a normoglycemic condition is responsible for approximately 80% of insulin mediated glucose uptake (Klip and Pâquet, 1990; DeFronzo and Tripathy, 2009). Consistent with the increases in circulating glucose and insulin levels in PD OVX, the expression of IRS1, a substrate of insulin receptor tyrosine kinase with a central role in the insulin signaling pathway and glucose uptake in SKM (Kovacs et al., 2003), was significantly lower in the SKM of PD OVX group compared to those in the OVX and OVX + E_2_ groups. We also observed a lower level of GLUT-4 expression in SKM of OVX groups compared to E_2_-treated groups, regardless of being OVX or PD OVX. In similar lines, Gorres et al. recently reported estrogen treatment improved insulin signaling and enhanced SKM glucose uptake in ovariectomized rats (Gorres et al., 2011).

An alternative insulin dependent pathway for GLUT-4 translocation to the membrane and increasing glucose transport is by the phosphorylation of AMPK (Gorres et al., 2011). Previous reports suggested that AMPK activation modulates glucose uptake and GLUT-4 translocation in SKM (Kurth-Kraczek et al., 1999; Fryer et al., 2000). There is substantial evidence suggesting that AMPK is dysregulated in animals and humans with T2D, and that AMPK activation (physiological or pharmacological or hormonal) could improve insulin sensitivity (Coughlan et al., 2014). E_2_ treatment is shown to increase AMPK activation in C2C12 myotubes (D’Eon et al., 2008). Accordingly, E_2_ treatment appears to enhance AMPK-α and p-AMPK-α in SKM of the PD OVX + E_2_ groups compared to other groups. D’Eon et al., reported that E_2_ promotes the partitioning of free fatty acids toward oxidation and away from triglyceride storage in SKM by upregulating the expression of peroxisome proliferation activator receptor-δ (PPAR-δ) and its downstream targets and also by directly and rapidly activating AMP-activated protein kinase (D’Eon et al., 2005). Although we did not attempt to elucidate the underlying mechanisms responsible for elevated p-AMPK-α following E_2_ treatment in the SKM, these data would appear to be in support of our metabolic data and E_2_ protection against weight gain, central adiposity, dyslipidemia, hyperglycemia and hyperinsulinemia.

Endothelial dysfunction is considered the initiating factor for vascular complication in diabetes (Bakker et al., 2009; Takenouchi et al., 2009; Eringa et al., 2013). Vehkavaara et al., reported that a higher glucose level, but not as high as in a diabetic state, could still lead to vascular dysfunction in male subjects (Vehkavaara et al., 1999). Accordingly, here we showed that the relaxation to ACh was significantly lower in PD OVX group compared to OVX rats. However, this impairment could be in part related to the higher level of contraction observed in PD OVX group. An intriguing observation of this study was that E_2_ replacement restored the impaired relaxation responses observed in PD OVX + E_2_, regardless of elevated contraction in this group (Figure 3; Table 1). Notably, ACh responses in MA of sham-operated PD group were significantly higher than that in PD OVX rats, suggesting that the ovarian hormone deficiency contributed to reduced mesenteric arterial relaxation in PD OVX group. Furthermore, E_2_ replacement enhanced the maximum vasorelaxation in MA of PD OVX + E_2_ rats to the level as observed in sham-operated PD rats (Supplementary Figure S2).

It has been shown that both NO-dependent and NO-independent mechanisms are involved in rat mesenteric arterial relaxation (Zygmunt et al., 1995; Zhang et al., 2012). However, the specific role of E_2_ on NO and NO-independent pathways in the prediabetic state is not clear. In the present study, we showed that the inhibition of COX metabolites by Indo plays only a minor role in ACh-induced relaxation of MA. Consistent with this are data demonstrating that COX metabolites have a less important role in the relaxation of smaller arteries, such as MA (Shimokawa et al., 1996). In the presence of Indo and L-NAME, the reduction in vasorelaxation is generally considered to represent the role of NO, and the remaining vasorelaxation to ACh is referred to as the L-NAME/Indo-insensitive component, or EDH-type relaxation (Feletou and Vanhoutte, 1988; Komori et al., 1988; Matchkov et al., 2012). This remaining ACh-induced vasorelaxation can be subsequently blocked by apamin and TRAM-34 (Doughty et al., 1999).

In all four experimental groups, the addition of L-NAME led to a further reduction of EDV (Figures 4A–D). However, the added effect of L-NAME in blunting ACh-mediated vasorelaxation was much more prominent and completely blocked the remaining relaxation responses to ACh in prediabetic arteries when compared with their respective controls (Figures 4C,D). Overall, these data suggest that in the OVX and OVX + E_2_ groups, both NO and EDH mediators contributed to the mesenteric arterial relaxation responses (Figures 4A,B; Table 2). This observation is in line with published reports by us and others who investigated ACh relaxation in MA of intact male and female rats (McCulloch and Randall, 1998; Zhang et al., 2012; Shaligram et al., 2020). An intriguing observation of this study is that in PD OVX group, NO is the only contributor to mesenteric arterial relaxation and EDH-type relaxation is absent, regardless of the presence or absence of E_2_. Therefore, the impairment of relaxation responses to ACh observed in the PD OVX rats may, in part, be due to loss of EDH-mediated relaxation, which is one of the major vasodilatory mediators in arteries in these groups. Furthermore, as shown in sham-operated PD group, it appears that the loss of EDH-type relaxation occurs as a result of the prediabetes status and not due to ovariectomy (Supplementary Figures S3D, E).

Notably, E_2_ replacement significantly increased the NO contribution to ACh responses in the PD OVX + E_2_ group when compared to the PD OVX group. It is, therefore, possible that observed enhanced relaxation in the PD OVX + E_2_ rats is partially mediated by the elevated contribution of NO in vascular relaxation in this group.

Previous reports have shown that the NO production may modulate the activity of the endothelium-derived hyperpolarizing factor (EDHF) pathway (Kilpatrick and Cocks, 1994; McCulloch et al., 1997). Thus, in the absence of basal NO production, the L-NAME-insensitive component of vasorelaxation is upregulated as suggested by Kilpatrick & Cocks (Kilpatrick and Cocks, 1994). This back-up mechanism may be of considerable importance in disease states where NO activity is impaired, and it has been proposed that EDHF (Bauersachs et al., 1996; Brandes et al., 2000; Juguilon et al., 2022) may serve as a backup vasodilator in situations associated with an altered bioavailability of NO. Nevertheless, we and others have reported a decrease in EDHF response in the mesenteric and renal arteries from diabetic animals (Fukao et al., 1997; De Vriese et al., 2000; Makino et al., 2000; Zhang et al., 2012). Here, we are demonstrating a shift of NO & EDH to NO only in ACh-induced EDV in prediabetic arteries. Our data suggest that NO and EDH are the primary mediator of ACh-induced vasorelaxation in normoglycemic but not in prediabetic arteries. Our previous studies also showed the NO & EDH-to-NO only shift in EDV in MA from streptozotocin (STZ)-induced diabetic rats (Zhang et al., 2012). While there are some limitations associated with the current experimental design to detect the possible cross talk between NOS inhibition and EDH enhancement pathway, our data suggest that the inhibition of NOS by L-NAME doesn’t lead to an increase in EDH importance in EDV of prediabetic arteries. Consistent with this interpretation is the observation of the potential loss of EDH contribution to ACh relaxation following NOS inhibition in the prediabetic arteries (Figures 4C,D).

Other mechanisms that could explain the improved vascular function of PD OVX + E_2_ might include factors such as enhanced sensitivity of MA to NO or decreased responses to vasoconstrictor agents such as PE. Here, we observed that vasorelaxation to SNP, an indicator of arterial response to NO, was clearly impaired in the PD OVX rats compared to the other experimental groups. Therefore, the impaired responses to ACh in PD OVX rats may, in part, occur at NO interaction with MA. However, it is important to note that in the current study SNP-induced vasorelaxation was measured in intact vessels (in the absence of NOS inhibitors). Therefore, we are not able to rule out the possible inhibitory effects of basal NO on SNP responses (Moncada et al., 1991; Parkington et al., 2002; Ralevic, 2002). An intriguing observation was that E_2_ replacement significantly increased the SNP-induced vasorelaxation in both OVX and PD OVX groups when compared to their respective controls. Furthermore, despite the difference in sensitivity of MA to NO in OVX and OVX + E_2_ groups, ACh responses were not different between these groups. This result could indicate the participation of additional vasorelaxant factors/mechanisms, other than NO, in the maintained ACh-induced responses. Accordingly, here, we demonstrated that both NO and EDH are the contributors to ACh-induced MA relaxation in the OVX and OVX + E_2_ groups. This theory is further supported by observation that in PD OVX models both ACh- and SNP-induced relaxation were impaired. This could be attributed to the loss of EDH contribution to the ACh responses as well as decreased NO signaling in the PD OVX groups. Along similar line with this theory is the study of Koeppen et al. who reported that NO/soluble guanyly cyclase (sGC)/cGMP is the major effector of SNP-induced acute dilations of murine resistance vessels (Koeppen et al., 2004). Other investigators have also provided the evidence that SNP-mediated vasorelaxation requires sGC upregulation and therefore mediated by NO (Lovren and Triggle, 2000; Cogolludo et al., 2001; Ruiz-Torres et al., 2009). There are also reports of decreased expression of sGC and impaired NO signaling in the vascular smooth muscle following ovariectomy (Stice et al., 2009; Lino et al., 2018). Clearly, we did not examine the underlying mechanisms of SNP-induced vasorelaxation in the current study, therefore, we cannot exclude the role of other factors besides tissue release of NO that may mediate SNP-induced vasorelaxation. Further studies are needed to elucidate the specific role of NO/sGC/cGMP in vascular function observed in our experimental groups.

Another mechanism that could explain the significant impairment of vasorelaxation responses in PD OVX group, as stated earlier, is the enhanced contractile responses to PE observed in this group compared to normoglycemic group (Figure 6; Table 4). Theoretically, the elevated PE responses in PD OVX rats, regardless of presence or absence of E_2_ (Figure 6; Table 4), may partially result from a decreased release of relaxing factors (NO and EDH), a decreased sensitivity to NO, a reduced NO bioavailability, or an enhanced release of contracting factors. In the present study, we measured the effects of L-NAME on CRC to PE. Since pretreatment with L-NAME potentiated the PE contractile responses to a comparable extent in the MA of all four groups (Supplementary Figure S6, inserted table, ΔAUC), this excludes diminished NO as the cause of the increased PE responsiveness in PD groups (Rahimian et al., 1997a, 2002; Dora et al., 1997, 2000; Zhang et al., 2012). Although the effects of SK_Ca_ and IK_Ca_ channel blockers together with L-NAME on the CRC to PE were not examined, the fact that EDH contribution to ACh vasrelaxation was lost in PD arteries (Figures 4C,D), suggesting that the diminished EDH may, in part, contribute to the increased contractility observed in PD arteries.

Here, we did not measure reactive oxygen species (ROS), but consistent with our working hypothesis are data demonstrating that expression levels of NOX-1 catalytic subunit of NADPH oxidase, the major source of superoxide in the vessel wall (Thompson et al., 2017) was significantly elevated in MA taken from PD OVX rats, irrespective of the presence or absence of E_2_. A previous report showed that ROS originated from NOX regulates PE-induced myosin light chain phosphorylation and induce contraction in rat tail arteries (Tsai and Jiang, 2010). In diabetes, superoxide production has been shown to play an important role in activating endothelium-derived contracting factors-mediated responses (Shi et al., 2007; Shi and Vanhoutte, 2008; Vanhoutte and Tang, 2008; Zhang et al., 2012). Overall, the increased NOX-1 expression in MA is in accordance with elevated contractile responses observed in PD groups. Notably, regardless of elevated PE responses in PD OVX + E_2_ group, the vasorelaxation responses were significantly improved in this group. This excludes reduced PE contractile responsiveness as the cause of increased vasorelaxation responses in the PD OVX + E_2_ group, suggesting the involvement of other mechanisms such as enhanced NO contribution to ACh responses and elevated NO interaction with MA in the PD OVX + E_2_ group.

In conclusion, this study is the first to report on the role of estrogen in protecting MA from early vascular dysfunction in prediabetic female UCD-T2DM rats, possibly *via* elevation of the NO contribution to vasorelaxation and its interaction with arteries as well as potential improvement in insulin signaling. Clearly, future studies are required to clarify the functional consequences of the estrogen effects of the elevated NO contribution to vasorelaxation in prediabetic female arteries.

Limitation of this study was that the normalization factor 0.9, established for the mesenteric arterial vessels, was not used in the current study. Although the final pressure was close to 13.3 kPa, it might not be exactly uniform in all the arterial segments.

## Data Availability

The original contributions presented in the study are included in the article/Supplementary Materials, further inquiries can be directed to the corresponding author.
