# Supplementary figures and images for: 17β-Estradiol Treatment Improves Acetylcholine-Induced Relaxation of Mesenteric Arteries in Ovariectomized UC Davis Type 2 Diabetes Mellitus Rats in Prediabetic State
Source: Front Physiol. 2022 Jun 17;13:900813. doi: 10.3389/fphys.2022.900813 (PMC9248973; doi:10.3389/fphys.2022.900813)

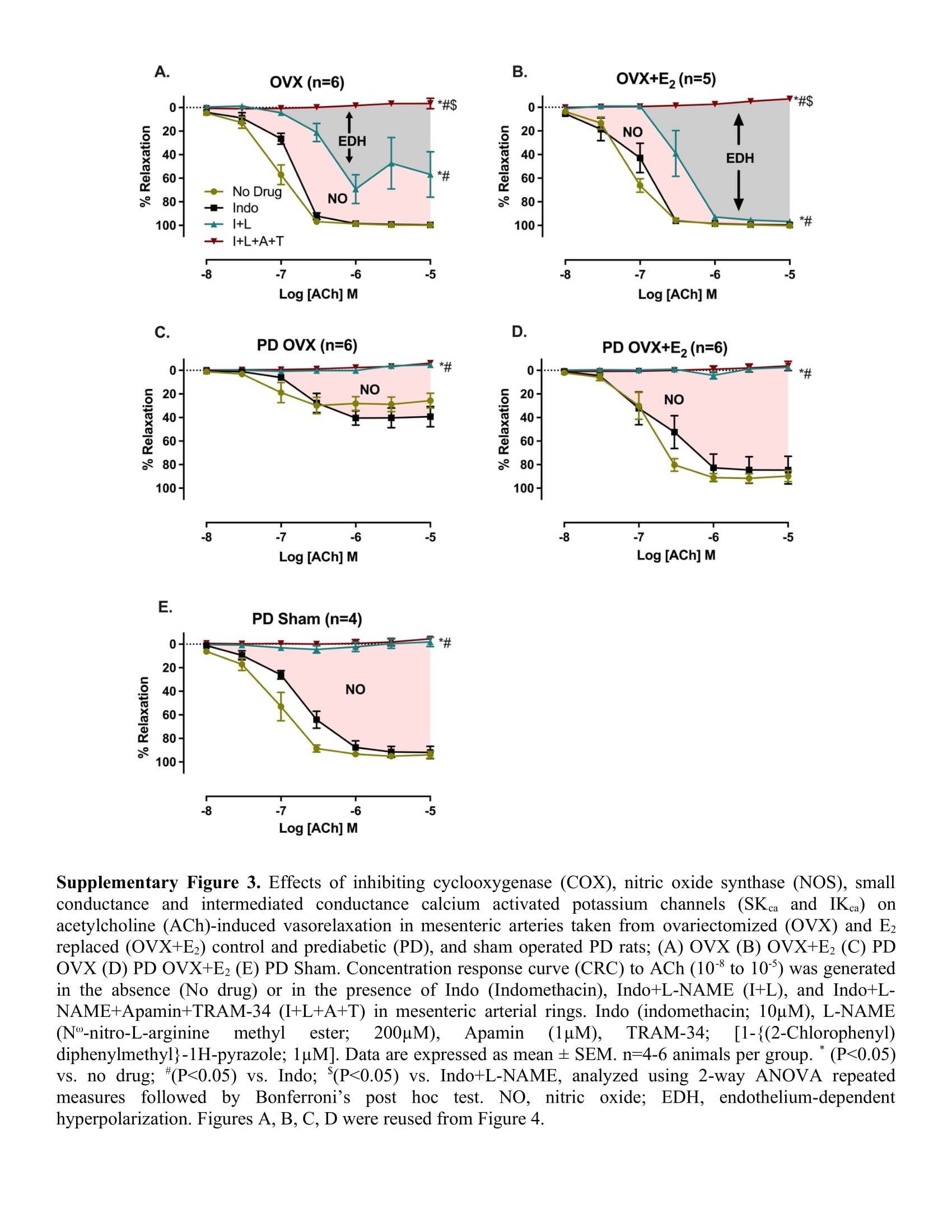

Supplement: Supplementary file 1 [file Image3.jpg]

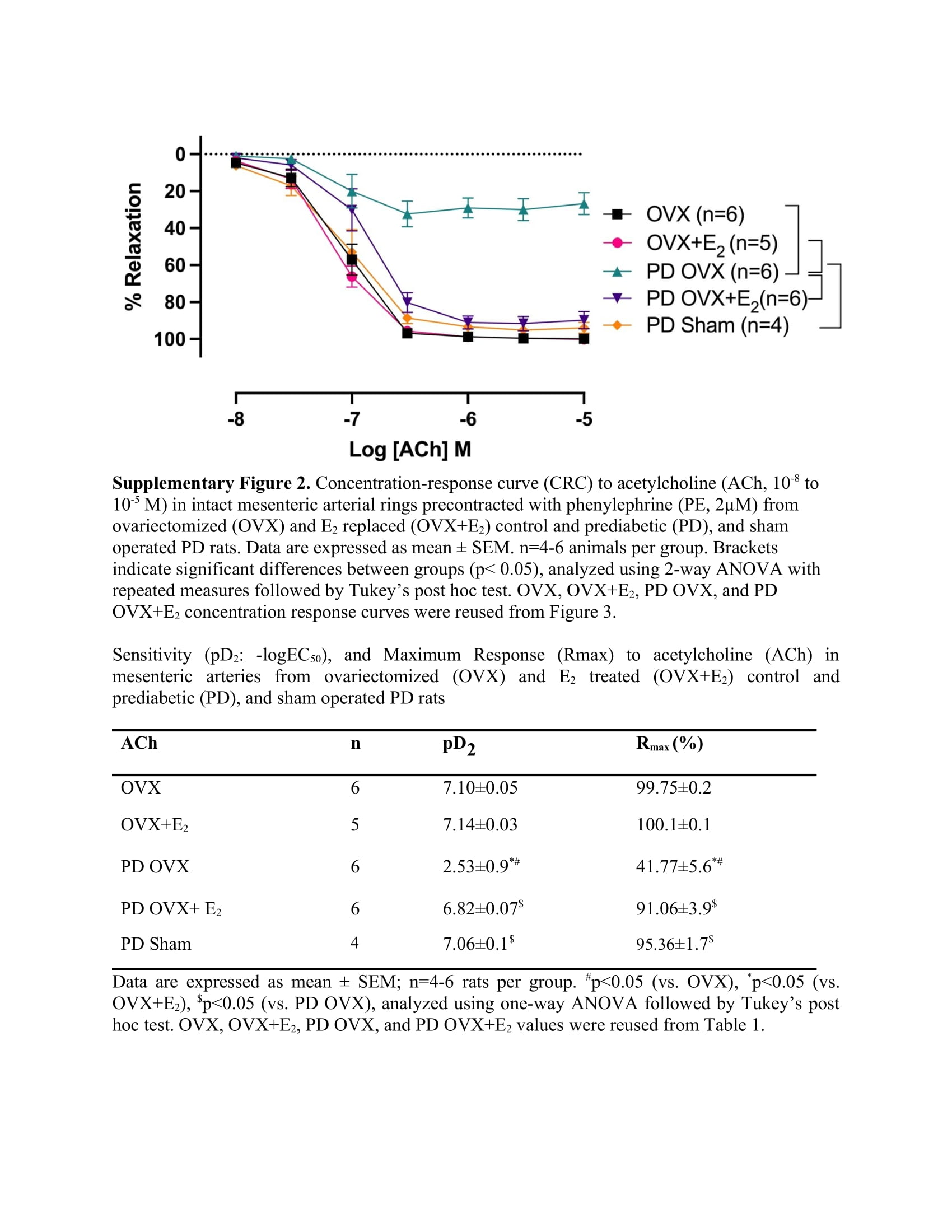

Supplement: Supplementary file 2 [file Image2.jpg]

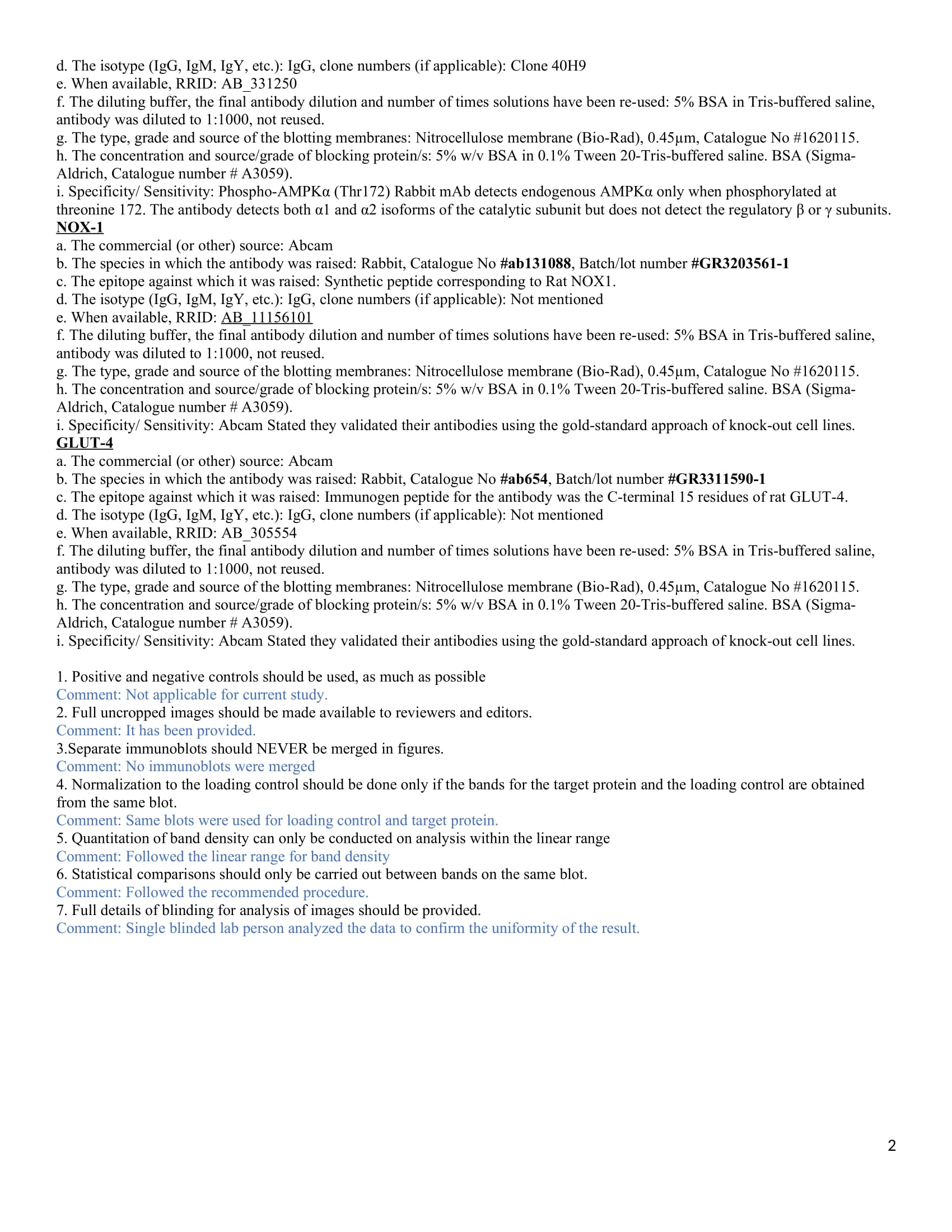

Supplement: Supplementary file 3 [file Image9.JPEG]

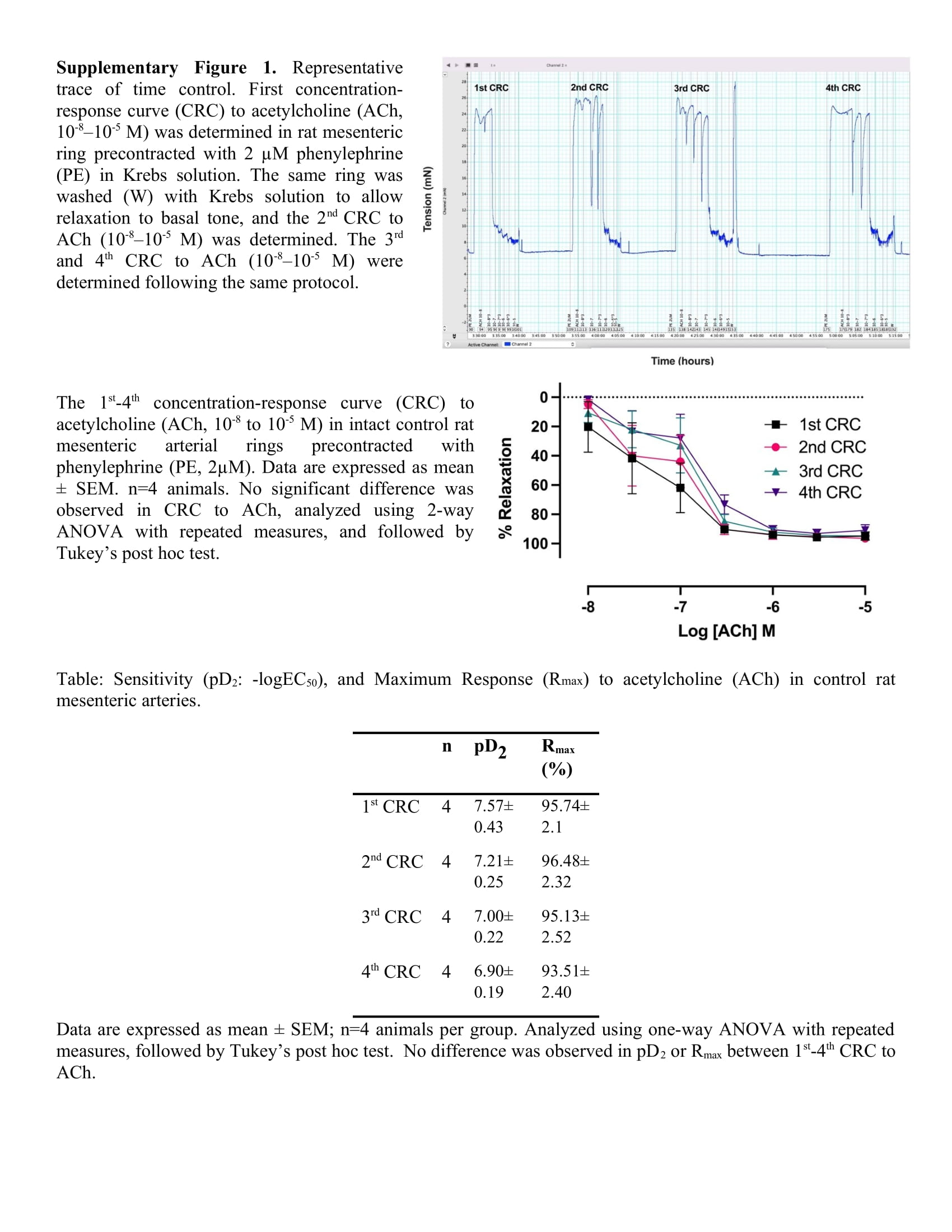

Supplement: Supplementary file 4 [file Image1.JPEG]

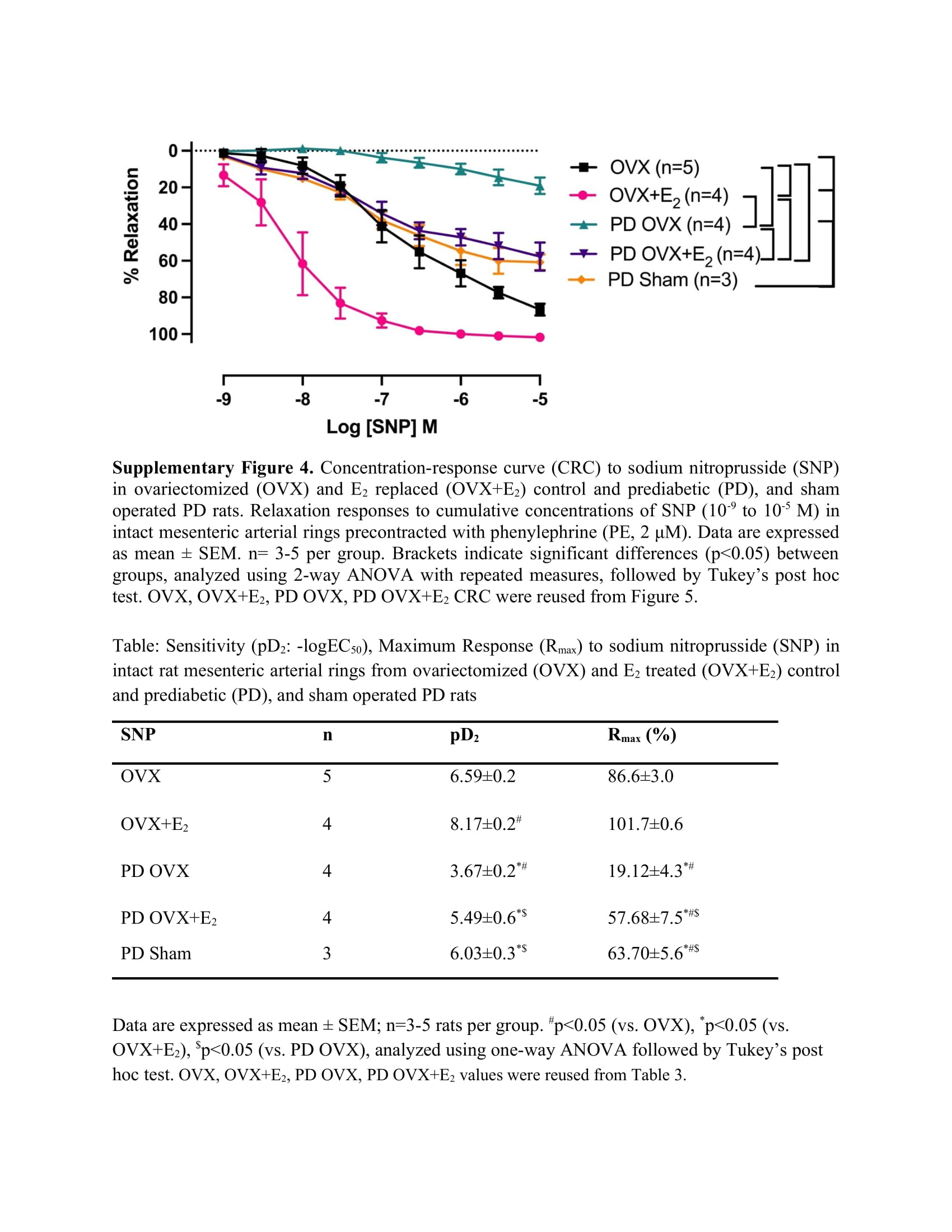

Supplement: Supplementary file 5 [file Image4.JPEG]

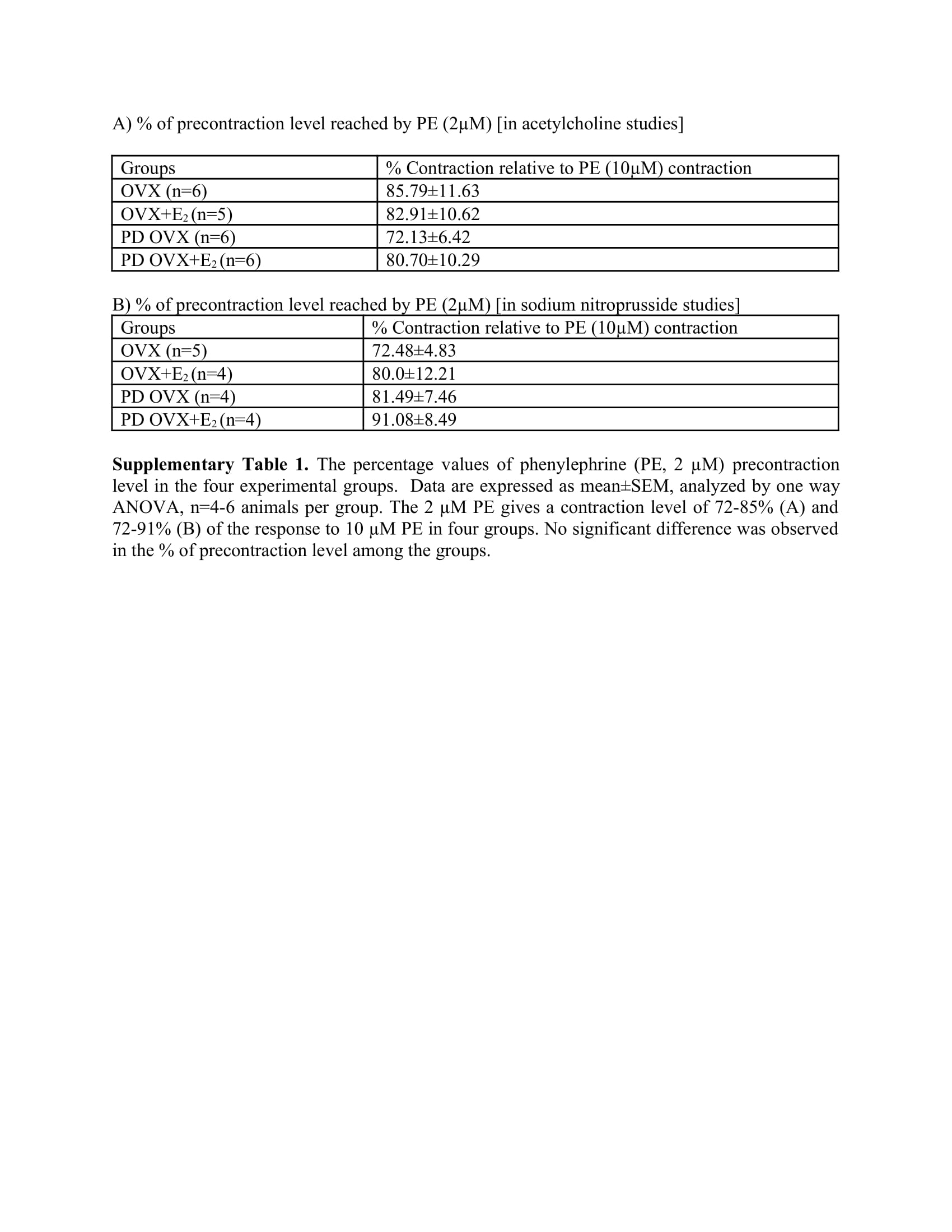

Supplement: Supplementary file 6 [file Image7.JPEG]

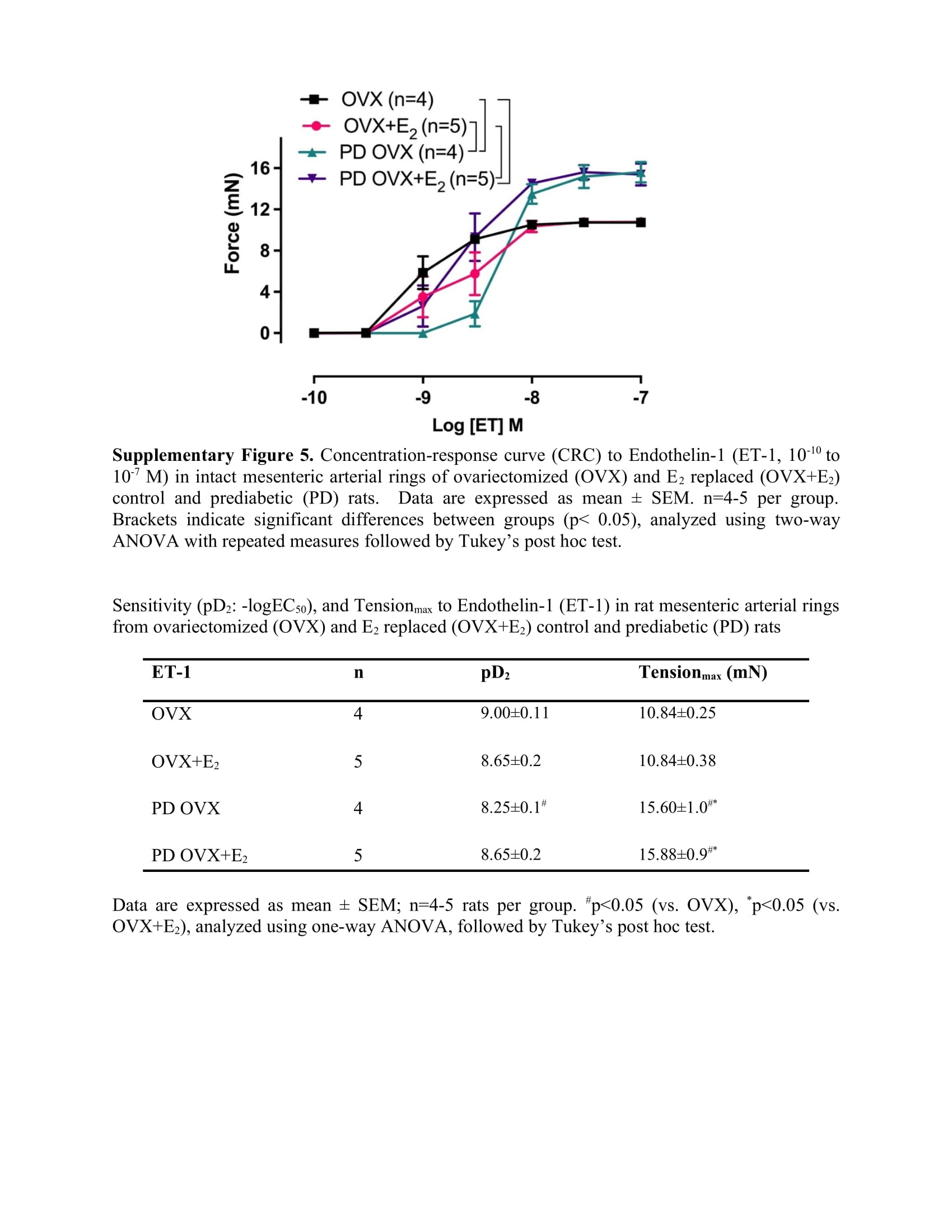

Supplement: Supplementary file 7 [file Image5.JPEG]

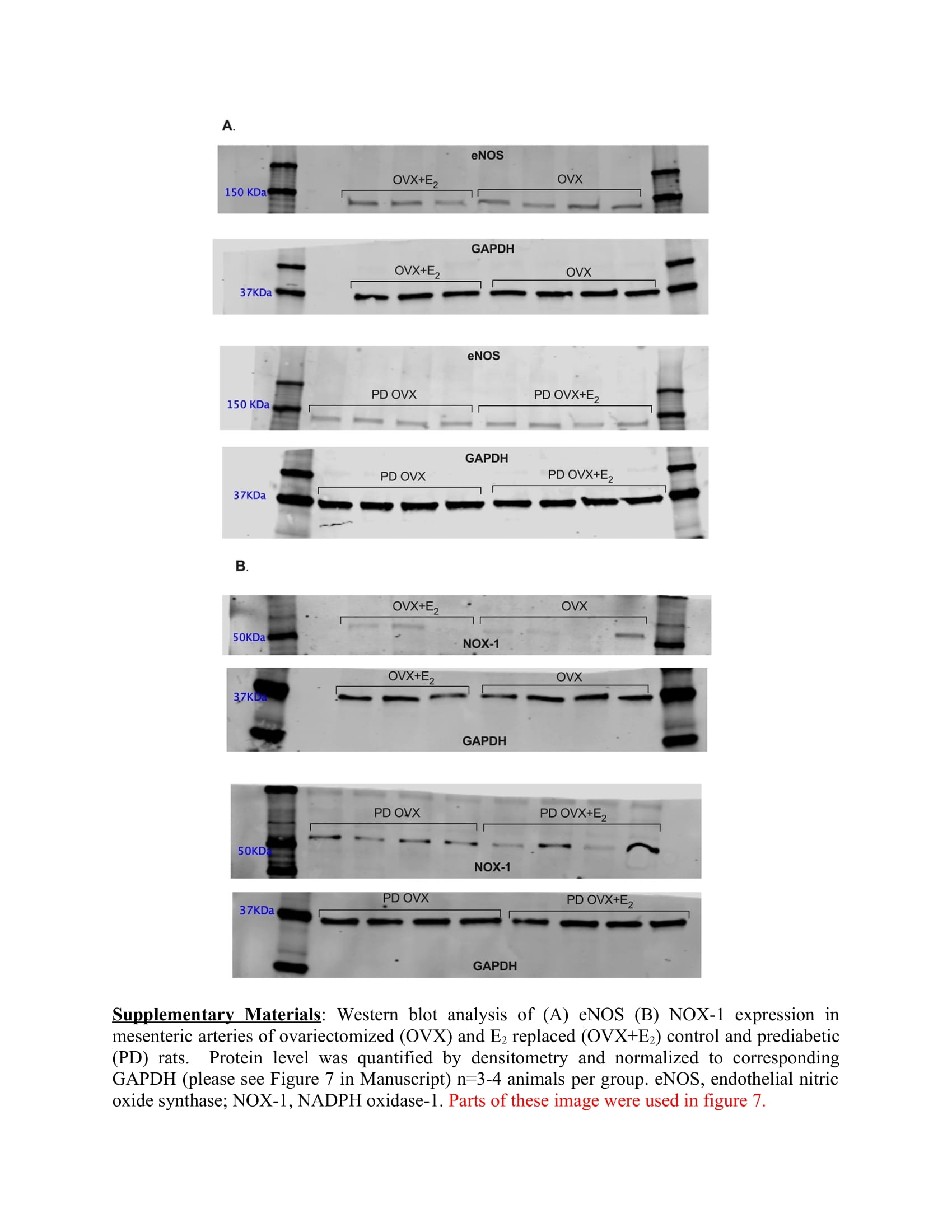

Supplement: Supplementary file 8 [file Image10.JPEG]

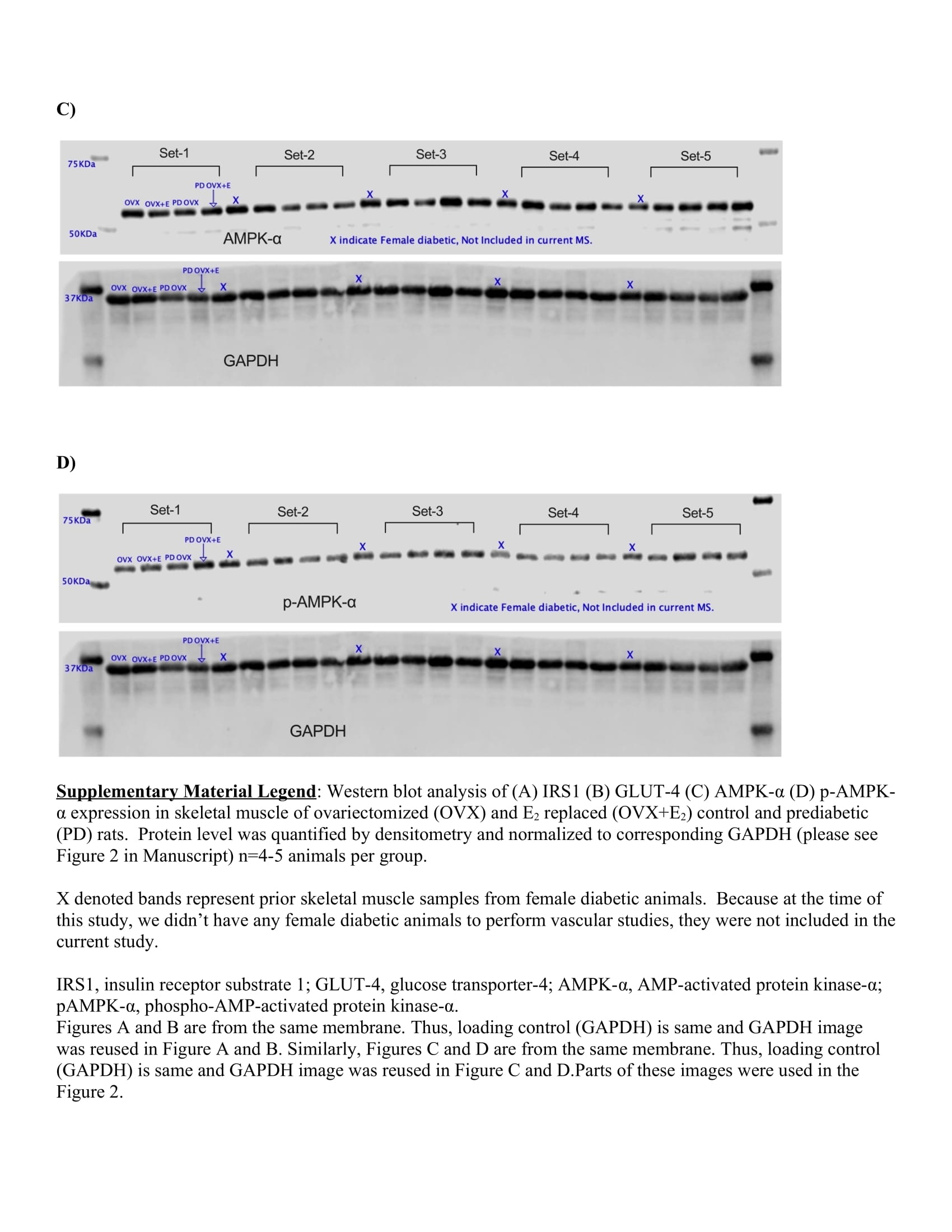

Supplement: Supplementary file 9 [file Image12.JPEG]

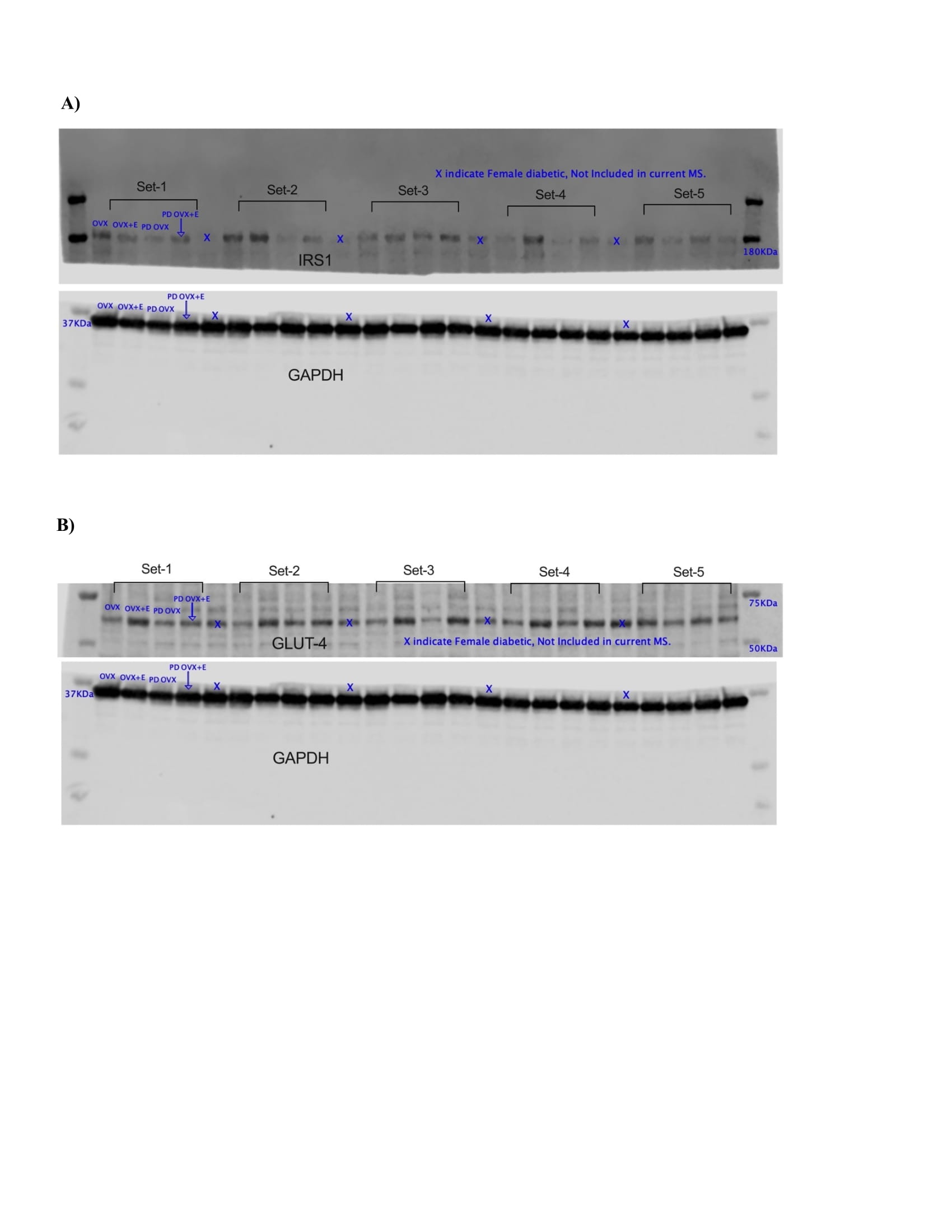

Supplement: Supplementary file 10 [file Image11.JPEG]

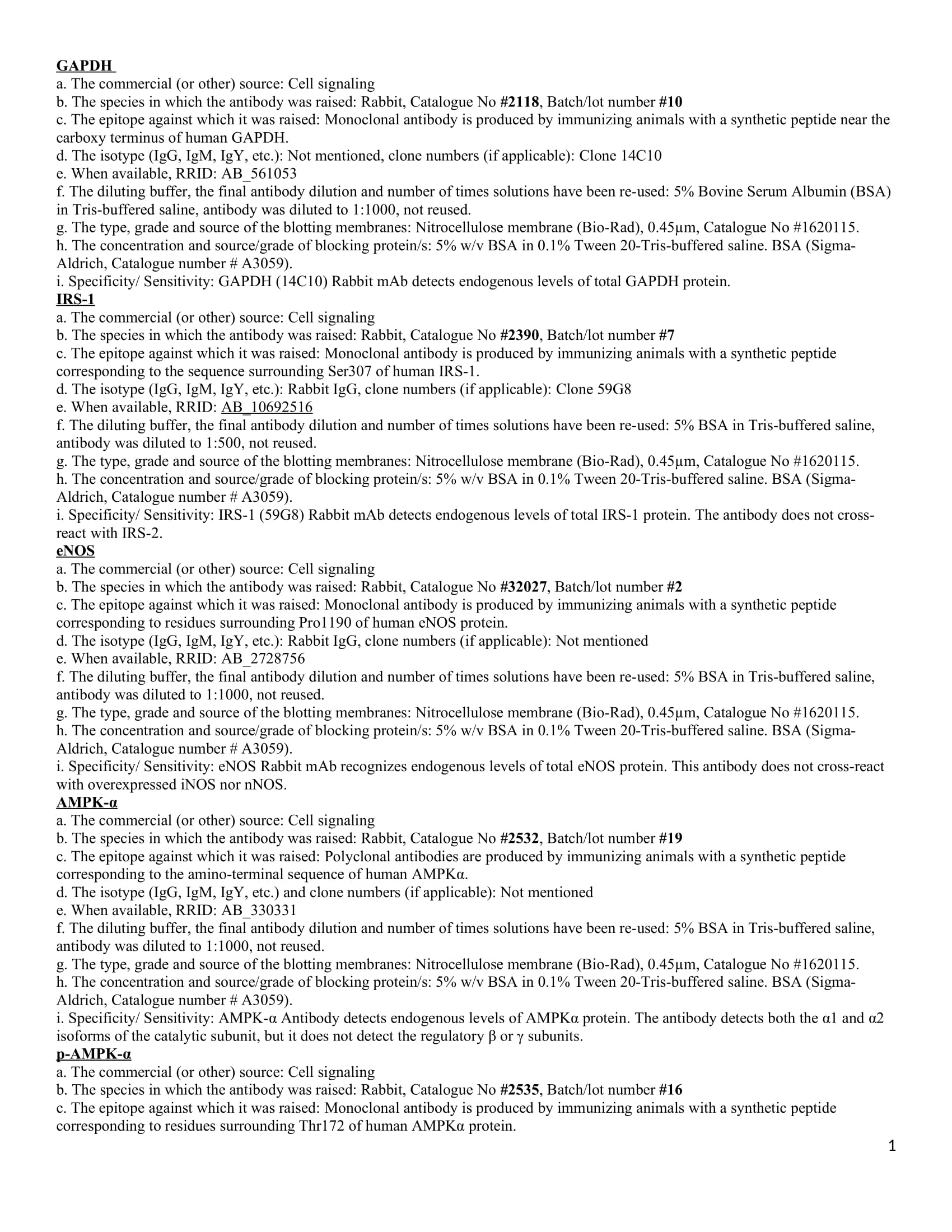

Supplement: Supplementary file 11 [file Image8.JPEG]

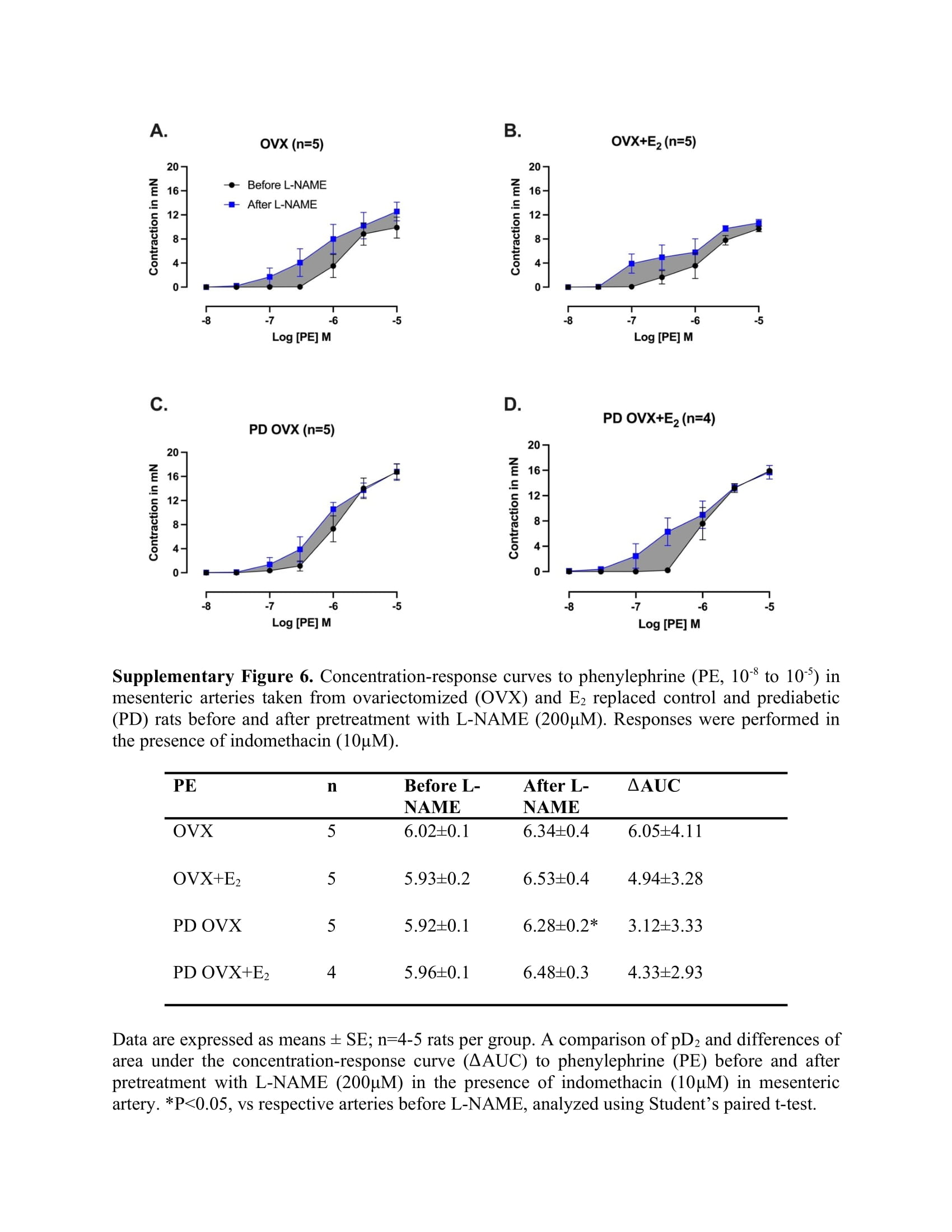

Supplement: Supplementary file 12 [file Image6.JPEG]
